# The parietal lobe evolution and the emergence of material culture in the human genus

**DOI:** 10.1007/s00429-022-02487-w

**Published:** 2022-04-22

**Authors:** Emiliano Bruner, Alexandra Battaglia-Mayer, Roberto Caminiti

**Affiliations:** 1 Centro Nacional de Investigación Sobre la Evolución Humana, Burgos, Spain; 2 Department of Physiology and Pharmacology, Sapienza Università di Roma, Roma, Italy; 3 Neuroscience and Behavior Laboratory, Istituto Italiano di Tecnologia (IIT), Roma, Italy

**Keywords:** Parietal cortex, Evolutionary anthropology, Comparative neuroanatomy, Tool use, Artifacts construction, Tool apraxia, Constructional apraxia

## Abstract

Traditional and new disciplines converge in suggesting that the parietal lobe underwent a considerable expansion during human evolution. Through the study of endocasts and shape analysis, *paleoneurology* has shown an increased globularity of the braincase and bulging of the parietal region in modern humans, as compared to other human species, including Neandertals. Cortical complexity increased in both the superior and inferior parietal lobules. Emerging fields bridging archaeology and neuroscience supply further evidence of the involvement of the parietal cortex in human-specific behaviors related to visuospatial capacity, technological integration, self-awareness, numerosity, mathematical reasoning and language. Here, we complement these inferences on the parietal lobe evolution, with results from more classical neuroscience disciplines, such as behavioral neurophysiology, functional neuroimaging, and brain lesions; and apply these to define the neural substrates and the role of the parietal lobes in the emergence of functions at the core of material culture, such as tool-making, tool use and constructional abilities.

## Introduction

There is consensus that the parietal lobe expanded substantially during the evolution of the genus *Homo*, and that this expansion is somehow associated with the sophisticated capacity to use tools and to manufacture the complex objects and artifacts necessary for foraging, defensive behavior, housing, and for manifold individual and collective daily activities. This perspective stems from the combination and cross-fertilization of experimental approaches, results, and models offered by different disciplines. These include evolutionary anthropology, rooted on the study of fossil records and living primates, and modern neuroscience, with special reference to comparative neuroanatomy, genetics, behavioral neurophysiology, and neuroimaging. Furthermore, emerging fields of investigation, such as neuroarchaeology, are providing intriguing experimental results concerning the items of behavior and neural activations associated with sensing and manipulating Paleolithic technology, while cognitive archaeology aims at interpreting this association from the perspective of current psychological theories.

Tool-making and use, and constructional activity are, indeed, a crucial topic in evolutionary anthropology, because technology is probably one of the most outstanding characters of the genus *Homo*. It is generally accepted that cultural evolution is associated with the encephalization process (for a discussion see Stout and Hecht 2017).

When dealing with studies on the relationship between tools and cognitive evolution, two main aspects must be considered. First, the term “tool” is often used in a very general meaning and without a clear biocultural definition. It has been proposed that tools should be characterized as obligatory elements of a cognitive and ecological niche, functionally integrated with the brain–body system, and associated with planned operational chains (Bruner and Gleeson 2019; Bruner 2021). According to this definition, we should distinguish between tool-using and object-using, with the former situation clearly recognized only in humans. The latter condition concerns the pragmatic use of objects as occasional resources not essential to the ecological or cognitive niche, without a profound integration into the body schemes, and through a direct use that does not involve a sequential productive chain with intermediate steps. This situation is likely the one generally found in non-human primates (NHPs), although conclusive evidence in this sense is still missing. Given these specific (and essential) premises, however, in this article, we will use the term tools in a general way, as commonly found in the literature. Second, most research was focused on tool-use and tool-making, but less attention has been paid to tool-sensing, which is actually the component behind the structural and functional integration of brain, body and technology (Bruner et al. 2018a, b). The neural pathways involved in making, using, and sensing a tool are necessarily part of a distributed system, but they also rely on different and independent networks. As such, these three components may have evolved independently, at different rates or with distinct combination of features.

Overall, as it will be discussed in the first part of this manuscript, the ability to manipulate materials and identify their structure and potential use as tools, together with constructional abilities, are hallmarks of human evolution. The development of this material culture, consisting in the capacity to learn from each other, has been the subject of intensive study and remains of significant interest not only in biological but also in philosophical sciences. According to the philosopher Henri Bergson (1907), prehistory and history suggest that it would be more appropriate to use, instead of *Homo sapiens*, the term “*Homo faber*,” who manifests his “intelligence” in the ability to transform the raw matter into artificial objects, understanding their possible use as “tools”. Furthermore, it has been suggested that these capacities and adaptations stay at the heart of the evolution of sociality and of a “social brain” (Boyd 2018).

Object manipulation, tool use, and constructive skills are intimately related cognitive visuomotor functions, which can be considered as a crucial building block of human cognition. Together with language and mathematical reasoning, this has been essential to make the human brain unique.

In humans, the structure of objects promotes inferences about their potential use as tools, while the use of familiar tools rests on memory of previous use. Object construction requires a visuospatial analysis of the available materials (raw matter, elementary building blocks, etc.) and a plan for assembling them through ordered movement sequences, based on the mental image of a model and/or on a physical copy-model. Such mental processes are absent in chimpanzees (Povinelli 2000), although this species can make and use simple tools and construct simple artifacts as nests. These mental processes are only present in humans, probably due to the expansion of the parietal lobe, to the inferior parietal cortex connectional asymmetries and to the emergence of hemispheric specialization across evolution (Cheng et al. 2021).

However, beyond the specific role that the parietal lobe might have in object construction, object manipulation and tool use, it is worth emphasizing the core role of posterior parietal cortex (PPC) in several visuomotor functions, whose integrity allows complex hand–object interactions. In fact, within the distributed parieto-frontal network (Battaglia-Mayer and Caminiti 2019) there are functional domains (Caminiti et al. 2015, 2017; Battaglia-Mayer and Caminiti 2018; Kaas et al. 2018) which are essential for accurate object manipulation, through appropriate visuomotor transformations, eye-hand coordination (Battaglia-Mayer et al. 2000; Battaglia-Mayer 2001), hand grasping (Jeannerod et al. 1995; Borra et al. 2017) and fine control of hand force (Ferrari-Toniolo et al. 2015), just to quote only some of the most relevant functions necessary when dealing with tools and objects.

## Parietal lobes and evolutionary anthropology

**Fig. 1:**
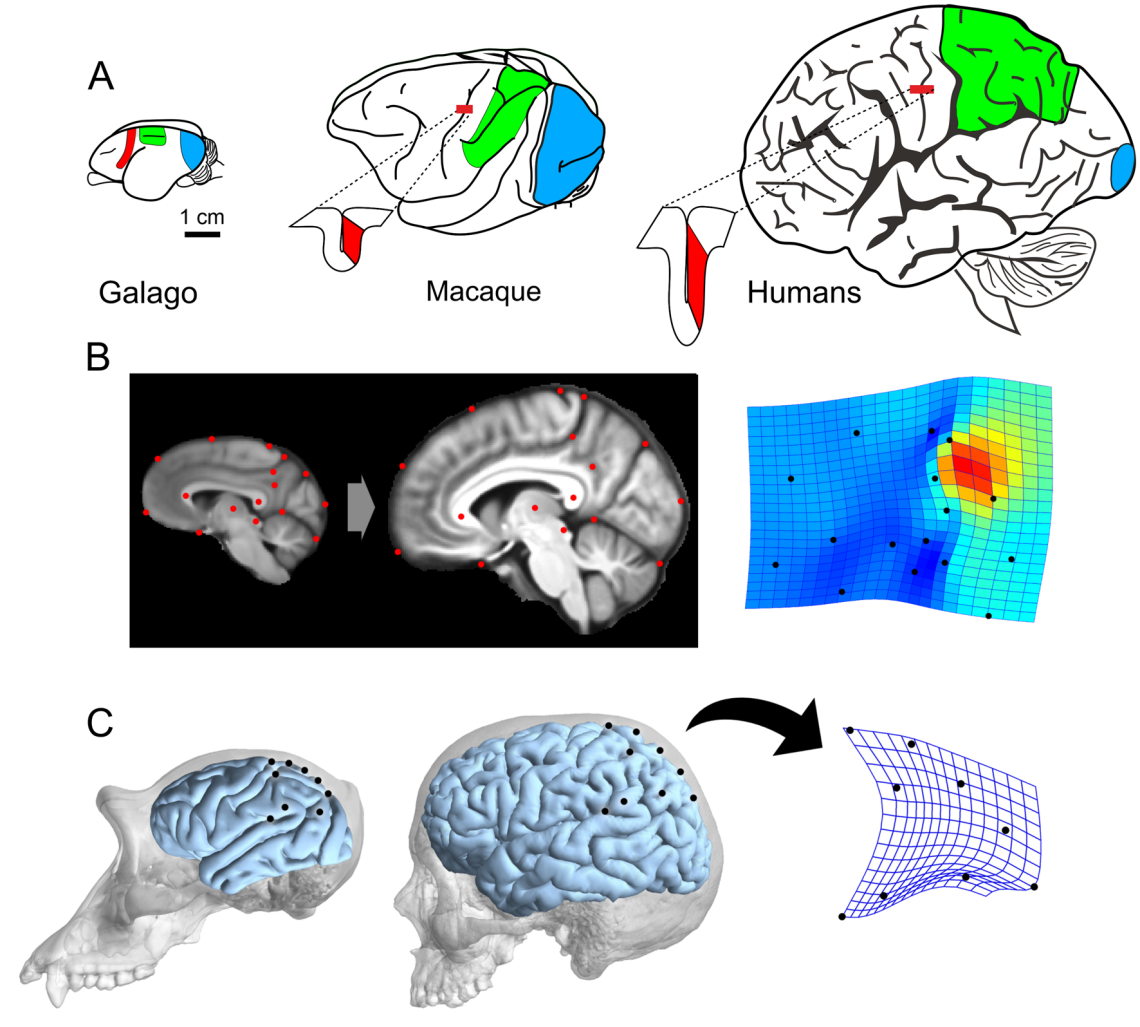
Expansion of parietal cortex in primates. **A** The brain figurines illustrate the expansion of parietal cortex (green), as compared to primary somatosensory (red) and visual cortex (blue), in prosimians (Galago), macaques, and humans. In macaques and humans, the insets visualize the location of the primary somatosensory cortex (red) in the posterior bank of the central sulcus (after Goldring and Krubitzer 2020). Humans have proportionally larger and more complex parietal cortex than non-human primates (human brain is not to scale). **B** Average MRI midsagittal brain templates of chimpanzees (left) and humans (right) are compared according to a geometric model using a set of landmarks (red dots). On the right, shape changes across brain regions are illustrated through a thin-plate spline deformation map (red: dilation; blue: compression; landmarks: black dots). The main spatial difference observed is associated with a disproportionally larger precuneus in our species (Bruner et al. 2017). **C** Comparing the main external landmarks (black dots) of the lateral aspect of the parietal lobe in chimps (left) and humans (right), human parietal shape is characterized by longer dorsal region and larger supramarginal cortex, as illustrated by the expansion grid on the right of the human parietal cortex (digital reconstructions by courtesy of Aida Gómez-Robles)

The parietal lobes underwent a remarkable expansion and specialization in primates in comparison with other mammals (Goldring and Krubitzer 2020; Fig. 1A). However, the homology between the different areas in different taxa is still a matter of debate, most of all when considering the complexity of PPC in humans (Zilles and Palomero-Gallagher 2001; Caminiti et al. 2015; Amunts and Zilles 2015). Morphometric comparisons (Fig. 1B, C) show that, in living humans, the precuneus, which occupies the medial wall of the superior parietal lobule (SPL), is much larger than in chimpanzees (Bruner et al. 2017). Furthermore, in humans, the intraparietal sulcus (IPS) is particularly developed, often markedly gyrified, and occasionally presenting several subsulci (Grefkes and Fink 2005), distinct from the IPS of chimpanzees and macaques. These results are in keeping with comparative data from macaques, which provide general evidence for hotspots of expansion located in the human association cortex, including the inferior parietal lobule (IPL; Van Essen and Dierker 2007). The IPL is highly specialized in core functions belonging to human species, such as social cognition and language (Binder et al. 2009; Bzdok et al. 2016; Graves et al. 2010). Indeed, there is no doubt that the human parietal cortex underwent an important evolutionary specialization, despite the attention devoted to this region by evolutionary and comparative studies (for comprehensive reviews, see Caminiti et al. 2015; Berlucchi and Vallar 2018; Caspers and Zilles 2018; Kaas et al. 2018; Palomero-Gallagher and Zilles 2018) is more recent, relative to other cerebral districts (Bruner 2018), such as prefrontal cortex (PFC; Avants et al. 2006; Passingham and Smaers 2014). Nonetheless, the phylogenetic correspondences of cortical areas are not particularly clear, and the evolutionary mechanisms behind the diversity of the parietal lobes in Primates are still hypothetical.

A major problem is homology, because the equivalence between human and non-human parietal areas is still under investigation. Much information is available for macaques and chimpanzees, but the relevance for human evolution is unclear. Living primates include hundreds of species and tens of genera, so caution is necessary to avoid generalizations. More importantly, living primates are not ancestral to the human genus, but represent parallel and independent evolutionary lineages. Therefore, their anatomical or physiological features cannot be interpreted as primitive traits, unless there is highly consistent information across several different taxa. In general, due to many limitations, a reliable phylogenetic analysis of the homology of the parietal cortical regions is at present, not feasible.

In evolutionary anthropology, the alternative to living species is the study of fossil specimens. In this case, we can detect (or at least suppose) ancestor-descendent changes, observing the process of evolution itself, instead of its product (Bruner 2019). However, also in this case there are important limitations: paleontological samples are generally very small, and inferences are based on few individuals represented only by scattered bony remains. Hence, the evolutionary information is valuable, but scanty. The contributions of other neuroscience disciplines will be therefore fundamental to offer hypotheses and novel cues, so as to provide the missing tessera of the mosaic. In this first section, we review the main evidence on parietal lobe evolution in the human genus, according to the available information on fossil species, namely integrating paleontological and archaeological data through three distinct fields that bridge anthropology and neuroscience: paleoneurology, neuroarchaeology, and cognitive archaeology.

### Parietal lobe: paleoneurology and fossils

Paleoneurology (or, more precisely, *paleoneurobiology*) deals with the study of brain anatomy in extinct species (Holloway et al. 2004; Bruner 2017). Brain form and cortical features are indirectly inferred by the information available from the endocranial cavity, including brain size, geometry, and proportions, sulcal morphology, or the imprints of the vascular network. Paleoneurology is strictly an anatomical field, integrated within the wider framework of functional craniology, and aimed at investigating the morphogenetic relationships between brain and braincase (Bruner 2015). In other words, it is a field strictly dealing with morphology, therefore cognitive inferences must be taken with caution.

Interestingly, in the early years of the discipline, Franz Weidenreich suggested that, looking at the endocranial casts of fossil human species, the region showing a noticeable degree of morphological evolution was not the frontal lobe (as expected by many scholars during the dawn of functional neuroanatomy, including phrenology and lobotomy), but instead the parietal lobe (Weidenreich 1941). His observation was then confirmed by a pioneering shape analysis, showing that the parietal surface was the most variable region in hominids and hominoids (Holloway 1981).

**Fig. 2:**
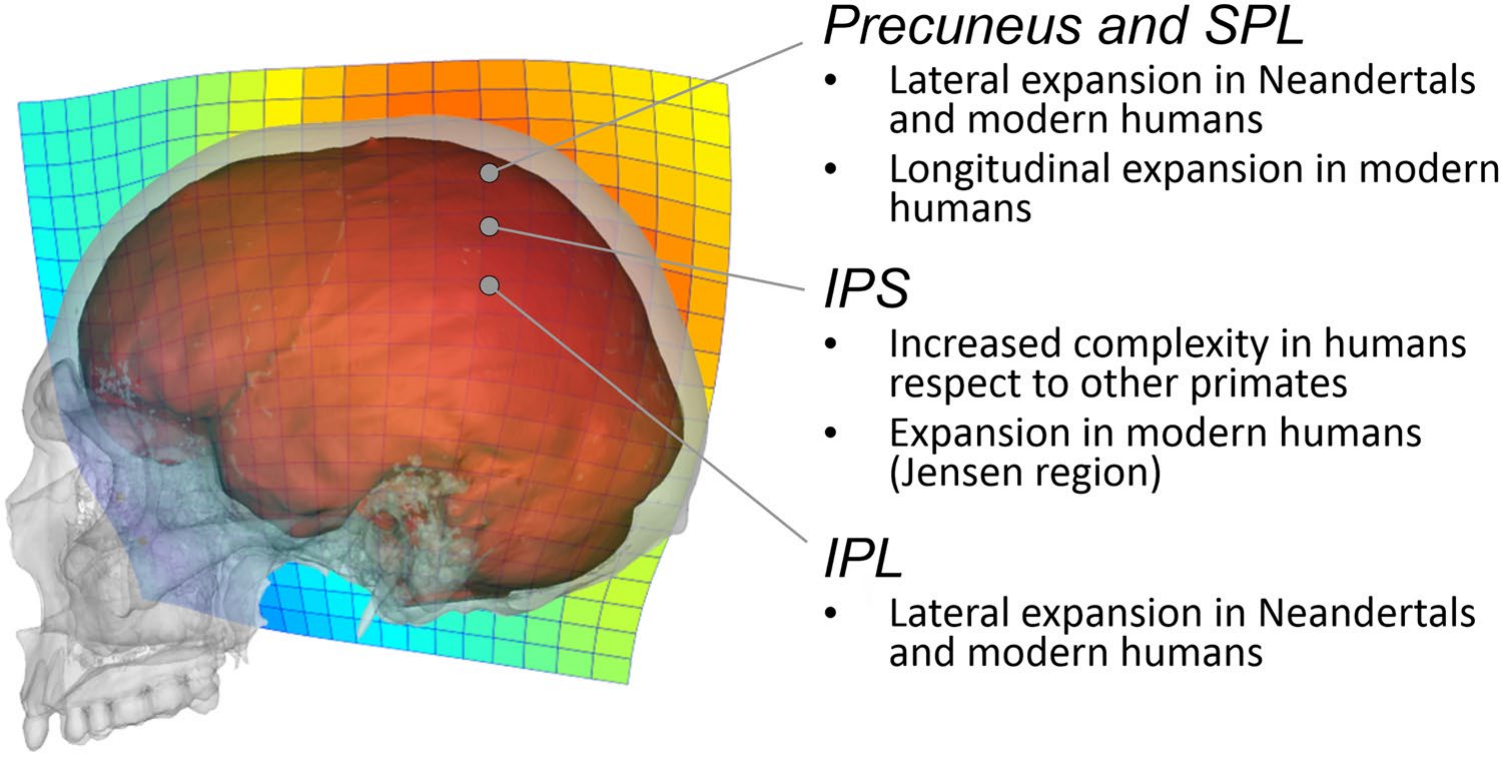
Our species, *Homo sapiens*, is characterized by large and bulging parietal bones (deformation grid after Bruner et al. 2014). Nonetheless, also the parietal lobes display a morphological expansion, in Neandertals and, to a major extent, in modern humans, at least when the cortical proportions are inferred from the sulcal traces left on the endocranial cavity (Bruner 2018)

A quick look at the fossil record suggests that the most noticeable change in the parietal bone occurs in our own species, *Homo sapiens*, in which it is definitely much larger than in any other human taxon (Bruner et al. 2011). Although the parietal bone covers several cortical regions beyond parietal cortex, its extension and growth are particularly associated with the morphology of the underlying parietal lobe (Ribas et al. 2006; Richtsmeier and Flaherty 2013; Bruner et al. 2015). All the other human species (including Neandertals, who shared with modern humans a similar range of brain size) display a much shorter and flatter parietal bone. Such expansion and bulging of the parietal region in our species are likely to be associated at least with two factors; namely, an increase of size due to cortical expansion (i.e., a brain factor), and a change of shape; namely, an increase in braincase globularity due to general architectural variations (i.e., a skull factor), like a reduced face and increased flexion of the cranial base, making the vault rounder (Fig. 2).

**Fig. 3:**
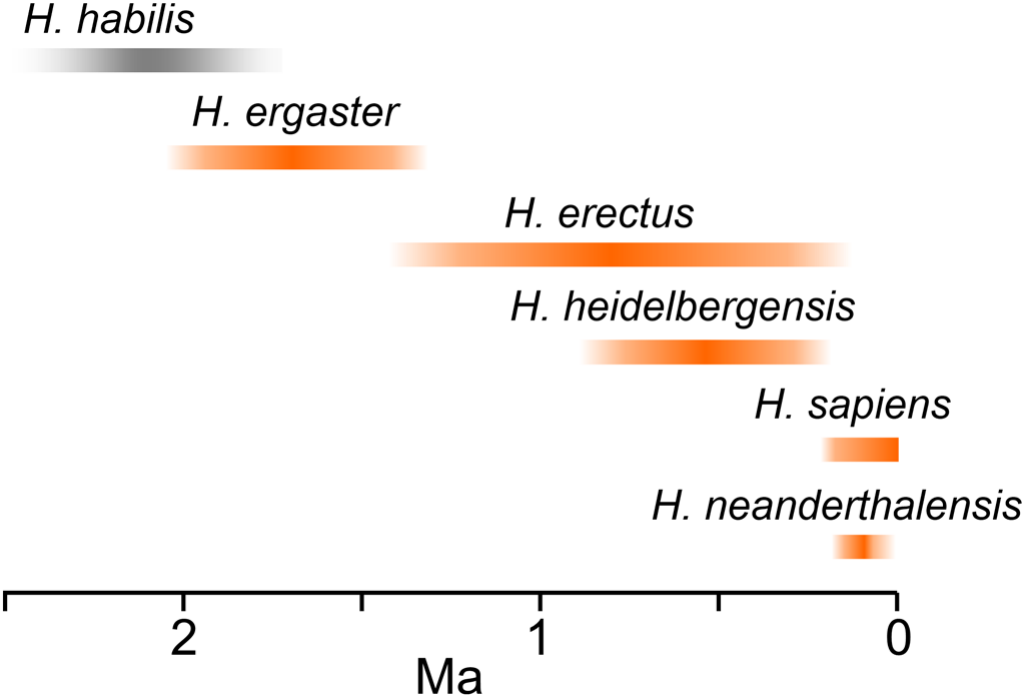
Although we ignore how many human species (i.e., belonging to the genus *Homo*) have existed in the last 2 million years, we have consistent paleoneurological evidence only for five taxa (orange shading). The species *Homo habilis (gray shading)*, interpreted as the first human species until the late 80s, has been successively questioned and, since then, left out from many phylogenetic hypotheses on the human genus. The paradigm of *H. habilis* was probably formed by specimens which belonged to distinct (two or three) species. These species can belong to the human genus, to *Australopithecus*, or to other genera which are scarcely known. Even those specimens possibly attributed to the genus *Homo*, can represent parallel human lineages, not ancestral to the later human species

However, an expansion of the parietal region in our species is also evidenced when the parietal *lobe* (instead of the parietal *bone*) is considered. Although the boundaries of the parietal cortex may be difficult to localize on endocranial casts, it is apparent that modern humans display a longer and larger parietal lobe, when compared with any other extinct human species (Bruner et al. 2003; Bruner 2004). Interestingly, the maximum brain width is localized at the temporo-parietal regions in the most archaic human species, at IPL in Neandertals, and at the SPL in modern humans (Bruner and Holloway 2010; Bruner et al. 2011). In early humans (Fig. 3) (*Homo ergaster/erectus*, after 2 million years ago, and *Homo heidelbergensis*, between 800,000 and 300,000 years ago), we can observe an expansion of the temporo-parietal cortex, but the dorsal parietal regions are short, flat and depressed. In Neandertals (say between 120,000 and 50,000 years ago) there is a remarkable enlargement of the IPL but also a slight lateral bulging of the dorsal parietal region. Early modern humans (200,000–100,000 years ago) had an overall brain morphology that was similar to Neandertals, but later populations (after 100,000 years ago) displayed a pronounced bulging of the dorsal parietal region (Bruner and Pearson 2013; Neubauer et al. 2018). Although a precise localization of these anatomical variations is difficult, a geometric model comparing Neandertals and modern humans suggested that, in our species, larger parietal lobes could be due to longer dorsal region (the posterior portion of the precuneus) and expansion of the middle parietal region, roughly corresponding to the Jensen sulcus, that is a branch of the intraparietal sulcus extending between the supramarginal (SMG, Brodmann 40) and angular (AG; Brodmann 40) gyri (Zlatkina and Petrides 2014; Wild et al. 2017). These characteristics are illustrated in a synthetic fashion in Fig. 2. Although a precise chronological assessment of these evolutionary changes is not feasible because of the paucity of the fossil record, this expansion of the parietal region in modern humans is roughly associated with increased tool complexity, graphical culture, projectile technology and enhanced haptic capacity, suggesting a specialization of the visuospatial integration system (Bruner and Lozano 2014, 2015).

### Brain functions and cognition in extinct humans

**Fig. 4:**
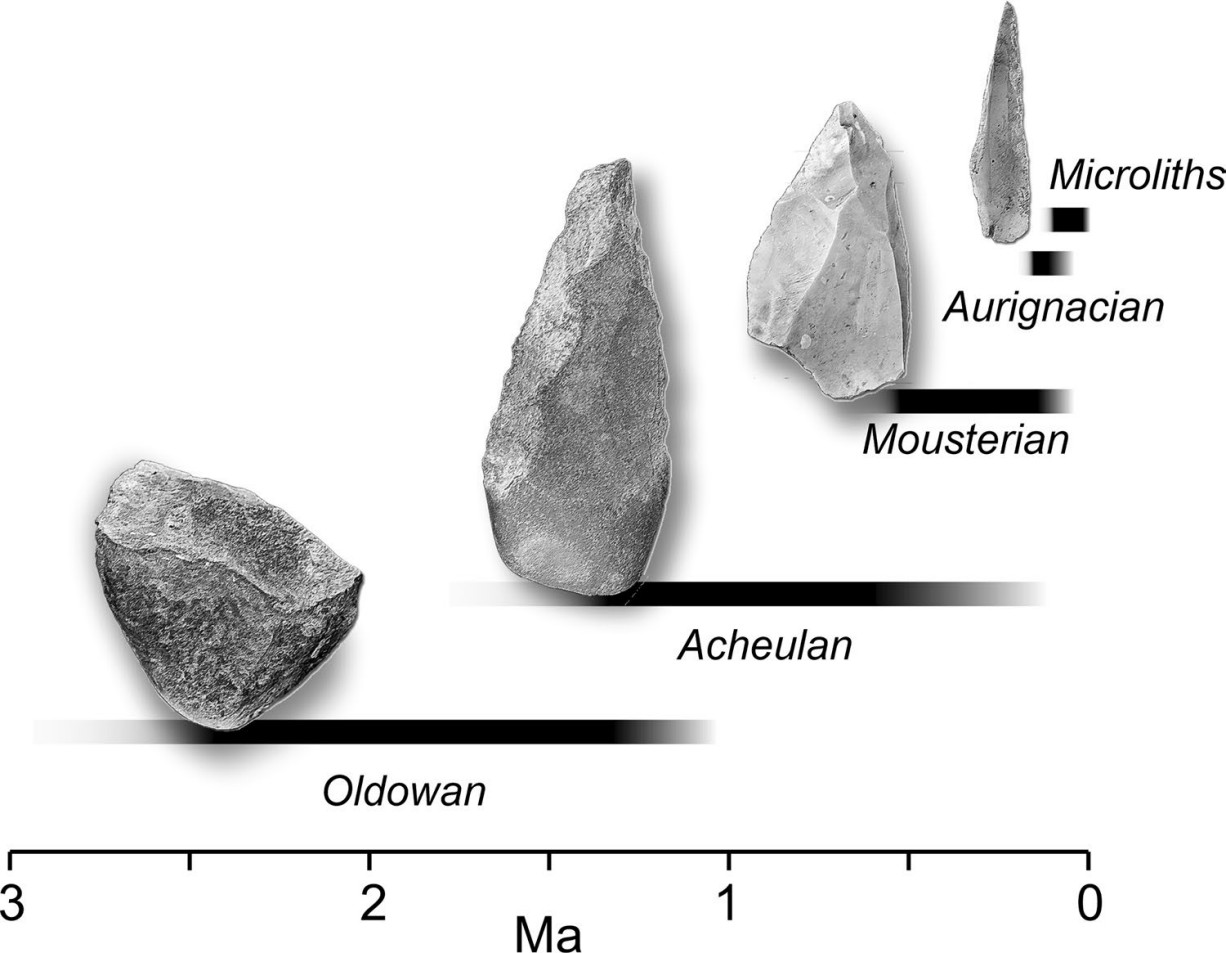
During human evolution, tools have become smaller and more complex, being handled largely by the whole hand in the earliest technological stages, then by the fingers, and finally with the fingertips. Although prehistoric archaeology has long relied on fixed typological models, it must be noticed that the variation of these tool types, both in terms of geography and chronology, is generally large, and their functions is not always clear. Some of these tools may have been handled, furthermore, through hafting. In general, later tools do not entirely replace but coexist with former and more archaic technologies (*Ma* million years)

*Neuroarchaeology* is a young field dealing with the use of methods in current neurobiology (mostly neuroimaging, such as PET) to investigate the brain activation during behavioral tasks consisting in making and using stone tools typical of the Lower Paleolithic (Stout and Hecht 2015). Tool-making and tool-use have been the most considered activities, because of the importance of lithic industry in the archaeological inferences on human cognition (Stout and Chaminade 2007; Stout et al. 2015). The earliest stone tool culture (Fig. 4) with a consistent archaeological record is the Oldowan, roughly dated to around 2 million years ago and associated with the origin of the human genus. It was based on a rough percussion of the stone, to achieve a cutting border or, possibly, basic flakes. Despite disagreements on its possible functions, there is evidence suggesting its use as a percussive tool. Functional imaging studies suggest that this operation largely relies on PPC activation (Stout et al. 2015). Around 1.5 million years ago, a different tool type, called Acheulean, was added to the general toolkit, in which the whole stone is knapped to obtain a roughly symmetric shape with a thin cutting edge all through the tool outline. This is possibly the main tool type associated with the human activity between 1.5 million years and 100,000 years, hence representing the most successful human technology to date, at least in term of duration. In this case, functional imaging (PET analysis) suggests that, beyond the parietal activation (specially the IPL), there is an important contribution of the prefrontal cortex (PFC), supposedly associated with behavioral planning and executive functions (Stout et al. 2015). The network underlying the visuomotor capacity associated with technology probably operates by integrating temporal, parietal and frontal functions, with humans displaying, in this sense, specialized connections between the main circuitry (present also in apes) and the SPL areas (Stout and Hecht 2017).

Another recent field integrating anthropology and neuroscience is *cognitive archaeology* (Coolidge et al. 2015; Wynn and Coolidge 2016). According to its principles, the items of behavior associated with the archaeological record (in this case, more precisely, with prehistoric archaeology) can be interpreted in the light of current theories in psychology. The field, to date, has been mainly developed on a theoretical and speculative ground, although some experimental and quantitative analyses have been recently proposed (Fedato et al. 2019; Silva-Gago et al. 2021, 2022). Also in cognitive archaeology, specific hypotheses have been suggested on the cognitive role of both the SPL and IPL. The SPL has been supposed to be associated with the complexity of the visuospatial behaviors, including brain-body-environment integration, technological integration, and self-awareness (Bruner and Iriki 2016; Bruner et al. 2018a, b, c). The IPL has been associated with language evolution, recursion, eye-hand-tool coordination and numerosity (Coolidge et al. 2011; Stout and Chaminade 2012; Coolidge and Overmann 2012). In general, most of these theories consider a distributed fronto-parietal system (Stout and Hecht 2017), taking into account a working memory model based on the integration of executive functions (PFC), a phonological loop (temporo-parietal cortex, TPC) and a visuospatial sketchpad (Wynn and Coolidge 2003; Coolidge and Wynn 2005). Interestingly, when we observe an early expansion of the SPL (that is, in Neandertals and early modern humans), large tools like chopper and handaxes, grasped with the whole hand, are substantially substituted by flakes (Mousterian industry), handled with the fingers. Lately, in association with the large parietal surface of modern humans, we found smaller tools like microliths, handled with the fingertips. Therefore, at least at the gross level of resolution available from this kind of evidence, it seems that a neural specialization co-evolved with a sensorimotor specialization.

It is worth noting that these major and conventional tool types are not strictly associated with a single human species. Any given species likely employed more tool types at once, and the beginning of a new technology does not match with the origin of a given species. Therefore, although in some cases generalizations are employed (*H. erectus* is often associated with Acheulean, *H. neanderthalensis* with Mousterian, *H. sapiens* with Aurignacian and so on), a one-to-one association between tool typology and human species is a superficial view that should be abandoned in both scientific and dissemination contexts.

### A cautionary note

In all these fields, a crucial limitation must be discussed. When dealing with extinct species, anatomy and behaviors associated with the fossil record are investigated through neurobiological or cognitive correlates in modern humans. Obviously, modern humans have modern minds, and inferences on brain anatomy and functions in extinct species are hence necessarily indirect. In experimental archaeology, similarly, modern humans are the ones making and using tools to investigate the behaviors of extinct human species. Namely, in all these disciplines, modern humans are used as a model for extinct humans. This should be interpreted as an implicit limitation, but not as a reason to reject the approach as a whole. First, every scientific field needs experiments and quantification, so as to move beyond mere theoretical speculations. In this case, Neandertal or *Homo erectus* subjects are simply not available for brain dissection, biomedical scanning or cognitive tests, and the only existing human species is our own. Second, the use of models is a common alternative in science, and this is not an exception for neuroarchaeology or cognitive archaeology, which uses models derived from cognitive sciences. In medicine and neuroscience, mice, cats, and macaques are often used as models to investigate human biology, when direct studies are not feasible. Considering these limitations, quantitative or experimental results from paleoneurology, neuroarchaeology and cognitive archaeology can add a scientific perspective to topics that, until now, were developed on a purely speculative basis, and this is a significant advancement.

### A prosthetic mind

In two million years, humans evolved from occasional to obligatory tool users; namely, a situation in which their ecological niche and cognitive processes are highly dependent on and intermingled with technology and culture (Shea 2017). The parietal cortex is deeply involved in the cognitive integration between brain, body, and environment, and hence a specialization of its areas is expected in *Homo sapiens*, who represents the most technological and symbolic species ever evolved so far. It has been hypothesized that neural and cognitive changes have increased the *prosthetic capacity* of our species, namely the possibility to integrate tools and symbols in our body schemes and cognitive machinery, outsourcing information processing to external peripheral elements (Malafouris 2010; Iriki and Taoka 2012; Bruner 2019, 2021). In this case, “mind” can be interpreted as a flow of information between brain, body, and material culture. The system formed by these three elements would be, therefore, the evolutionary unit undergoing selection and adaptation. According to these theories on cognitive extension, it is mandatory to investigate in detail the capacity to integrate tools into the brain schemes through the body interface. An increase in this capacity could have been a key evolutionary adaptation of our genus, in particular, of our own species.

### Tool use and object construction 2 million years after

In this section, we will discuss studies supporting the hypothesis that the unique ability of humans to understand causal relationships between available materials and their use as tools is dependent on the expansion and/or specialization of certain parietal areas near the IPS, in the anterior part of the supramarginal gyrus (SMG), while constructional abilities probably rest on the specialization of more posterior parietal and dorsolateral prefrontal (dlPFC) areas and on their distributed systems.

When studying tool use in animals, it is worth mentioning the observation by Hansell and Ruxton (2008) concerning the arbitrary separation between abilities related to tool-use and construction behavior in animals. Such separation resulted in a detrimental overestimation of the role of natural tool use, as compared to much more efficient anatomical adaptions. From this perspective, tool use would be uncommon in non-human animals not as result of constraints imposed by limited cognitive abilities, but because anatomical adaptations are by far more advantageous in evolution. As an example, the flexibility and adaptability to different situations when building nests (as in birds and in chimpanzees) suggest that constructional abilities involve cognitive processes that should not be underestimated when compared to those required in tool-users. However, it is also important to stress that natural multifunctional constructions, such as bird nests, often resulting from a tradeoff between conflicting needs (Mainwaring et al. 2014), are assembled without apparent causal understanding of the final goal and are rather the outcome of stereotyped action sequences (Collias 1964; Hansell 2000, 2005). In the invertebrate world, this applies also to spider webs and termite mounds, which are very complex artifacts apparently built without any “mental” representation of the goal (Gould and Gould 2012).

In humans, constructional praxis is a crucial ability of the brain for designing, drawing, composing mosaics, and creating complex structures, altogether referred to as artifacts, and can be recognized very early in life in children’s spontaneous play with blocks (Hirsch 1996). Constructional activities have been extensively used to study the development of spatial knowledge in children, as well as in apes, such as chimpanzees and bonobos. In this part of the manuscript, we will focus on these fundamental skills in non-human primates (NHPs) and humans, by stressing however that, to our knowledge, tool use and its neural bases have never been studied in the context of constructional behavior, for instance, when using a screwdriver to assemble a chair starting from its elementary component parts.

## Neuropsychological studies in humans: a brief overview on apraxia

The neural bases of tool use and constructive abilities have first received attention because of the dramatic consequences of their disorders in brain lesion patients, namely *tool apraxia* (TA) and *constructional apraxia* (CA), both affecting daily work, manufacturing, and a constellation of other activities, with significant social costs (see Krakauer and Carmichael 2017). These disorders have been recognized more than a century ago (Wilson 1908; Kleist 1934; Mayer-Gross 1935; Critchley 1953; Hécaen and Assal 1970) and can be conceptualized within the more general frame of the disturbances of praxis, that is of *apraxia* (Liepmann 1905, 1920)*.* Apraxia consists in the “inability to perform certain purposive actions, with conservation of motility, sensation and coordination” (Wilson 1908), and comes in several forms. Patients suffering from *ideational apraxia* (Liepmann 1920) fail to perform a complex action consisting in movement sequences needed, for instance, to fold a piece of paper and place it inside an envelope. In *ideomotor apraxia* (Liepmann 1920), patients cannot execute a familiar action on verbal command or by imitation. In both forms, the difficulty refers to reproducing previously well-learned motor skills, whereas in CA, the difficulty is with reproducing visual copy-objects and figures, such as geometrical drawings (cubes, rectangles, etc.) or complex images, such as the Rey figures (for a comprehensive review, see Gainotti and Troiano 2018).

In an initial attempt to identify the neuroanatomical substrates of different forms of apraxia, Liepmann (1920) emphasized the role of left parietal lesions. He attributed these disorders to a disconnection syndrome disrupting the information flow from posterior brain regions to motor cortex, so as to compromise the transformation of mental images into motor commands. More than a century later, apraxia is found in about 50% of patients with stroke predominantly in the left parietal cortex and affects action of both limbs (Krakauer and Carmichael 2017). However, also frontal lesions cause apraxic disorders, especially after stroke in the left hemisphere (Donkervoort et al. 2000). Apraxia has been regarded as the cognitive aspect of motor behavior (Goldenberg 2014), an intriguing perspective for its implications on the modular nature of the brain action systems.

In TA, patients can identify an object and remember the purpose it has been made for but fail to use it as a tool. Morlaàs (1928) termed this disorder “*agnosia of utilization*”. After decades of neglect, this issue was revitalized by De Renzi and Lucchelli (1988), who wrote: “The cognitive deficit […] concerns the ability to gain access to the semantic repository where the multiple features defining an object are stored, among which there is the way it must be used”. In the neuropsychological literature, two main views have characterized the study of TA. The first holds that TA consists in a disorder of special aspects of the semantic memory concerning knowledge about tool use, therefore in a disorder related to the planning of motor parameters necessary to grasp and manipulate a tool, the second focuses on a disorder of mechanical problem solving (for a comprehensive review, see Maravita and Romano 2018).

The latter perspective was proposed by Goldenberg and Hagmann (1998), who examined brain damaged patients in a study that required to select the tool most appropriate to solve a novel task, and contrasted retrieval of instructions from semantic memory with direct inference of structure from function, since the latter “[…] enable subjects to use unfamiliar tools and to detect alternative use of familiar one”. They considered this property as the basis of mechanical problem solving. While right damaged patients (RDPs) were impaired in the use but not in the selection of novel tools, the opposite was found for left damaged patients (LDPs), who in addition were impaired in the use and in pantomimes of familiar objects. Later, Goldenberg and Spatt (2009) performed an MRI voxelwise study of LDPs in different aspects of tool use, consisting in *functional association* (such as retrieval of functional knowledge from semantic memory), *mechanical problem solving* (use of novel, ad hoc made tools for tasks never performed before), use of *common tools* and objects. Frontal premotor patients performed poorly on all three tests, while parietal patients were impaired in both the selection and use of novel and common tools. The authors concluded that the PPC contains a representation of the general principles of tool use and contributes to the categorical apprehension (see also Goldenberg 2008) of the spatial interactions between multiple objects and multiple parts of an object, a process regarded as a necessary link in the chain of events leading from grip formation to tool manipulation and use. In this view, PPC would play a lesser role in the specification of hand geometry and hand movement necessary for tool use.

Taking a different perspective, in a study of *ideomotor apraxia* Buxbaum et al. (2007) found that patients with left IPL damage were preferentially impaired when producing and imitating object-related, hence transitive, gestures, rather than with symbolic intransitive gestures non involving objects. On the contrary, patients with bilateral damage of the fronto-parietal system, due to cortico-basal degeneration (CBD), did not show such difference. Furthermore, the most impaired component in patients with cerebrovascular accident was the hand posture of transitive gestures, which instead was only mildly impaired in patients with CBD. Finally, the latter were impaired in transitive hand posture, at variance from the parieto-frontal lesion that produced an opposite disorder. According to the authors, these observations are consistent with a model where encoding gestures using postural and movement-related information for manipulation of skilled familiar objects resides in the IPL. A more recent study by the same group (Buxbaum et al. 2014), building on the evidence accumulated in the last 15 years (see below) on the bilateral distributed nature of the tool use system (involving not only parietal, but also temporal and fontal regions) performed voxel-based lesion-symptom mapping from 71 stroke LDPs. Three types of actions were tested, gestures prompted by an observed tool, tool specific gestures imitation, imitation of meaningless gestures. In such a way, two out of three gesture types were tool-related, while two of three were imitative, favoring the study of common and different features in a pairwise fashion. The study of postural and kinematic accuracy, such as hand/arm position, amplitude and timing of gestures were also scored separately. Lesions in the left posterior temporal gyrus were associated with impaired postural components in both the tool-related and gesture tasks, while lesions in the inferior parietal and frontal cortex were associated with poor kinematic performance of all gesture tasks. Thus, according to this “componential neuroanatomical model”, the kinematic aspects are critical for imitation of meaningless gestures, while pantomimed gestures of viewed tools require representations of tool action and posture, with both capacities impacting on tool-related action imitation. The authors’ conclusion was that a gradual coordinates transformation occurs in the temporo-parietal interplay, where the representation of tools in visual motion coordinates is the first step toward a transformation of information in intrinsic coordinates. In this view, the IPL would encode motor plans from the analysis of the relative position of the body parts whose dynamic changes are necessary for shaping the action goal, therefore in intrinsic coordinates. This process relies on precise processing of somatosensory information.

Beyond the body and neural reconfiguration associated with tool use, it is worth noting that a first consequence of haptic stimulation concerns the emotional response due to a sensorial change. Even simple Paleolithic stone tools can exert changes in the levels of attention and arousal during naïve haptic exploration, activating a specific electrophysiological response when the body balance is altered by a potential technological extension (Fedato et al. 2019, 2020).

In conclusion, the neural underpinning of tool use, as seen from contemporary neuropsychology, remains a debated issue that will certainly provide interesting new results.

## Behavioral and neurophysiological substrates of tool use in non-human primates

From a behavioral point of view, one of the most representative forms of tool-use in chimpanzees in the wild is termite fishing, which consists in selecting and using a long thin and flexible stick inserted in the opening of a termite mound, as first documented by Jane Van Lawick-Goodall (1968). There is also conclusive evidence on the aptitude by capuchin (Visalberghi and Trinca 1989; Visalberghi and Limongelli 1994; Visalberghi and Tomasello 1998) and macaque monkeys (Tan et al. 2015) to crack nuts and oysters, respectively, after appropriate selection of stones used as tools. Sexual differences have also been reported in the latter species in stone tool use (Gumert et al. 2011). Although tool use can be learnt from observation by young unexperienced monkeys, and therefore socially transmitted, only humans continuously use, improve, and develop new and more complex and elaborated tools (Osiurak et al. 2010).

In apes, there is evidence of tool use, both in the wild (see Goodall 1986) and captivity (Visalberghi and Tomasello 1998), based on forms of associative learning, rather than on the causal understanding of the physical forces involved. These authors concluded that “apes show some possible signs of understanding the causal relations involved in tool use”. A more radical conclusion was reached by Povinelli (2000) through series of imaginative experiments designed to explore the chimpanzee’s causal reasoning during tool use, in particular their abilities to anticipate the causal relations between the tool features, the goal object, and the substrate (environmental aspects) on which the tool was used. In the laboratory setting, chimps were exposed to different options concerning tools to use (e.g., rake with broken vs intact handle, or regular vs inverted rake), and substrates on which to operate (table with or without holes, within which the food would necessarily fall after rake pulling). Whilst the chimpanzees had no difficulties to opt for intact rakes vs broken ones, correct responses above chance level were not observed when the animals were asked to choose between regular vs inverted (‘ineffective’) rakes. When exposed to two different tables, one with holes and one with painted ‘fake’ holes, they pulled the rakes regardless of the nature of the table surface. In a further study Povinelli (2000) analyzed tool use in chimpanzees during termite-fishing, by focusing on the ability to master the form-fitting relationships, therefore the spatial understanding necessary to insert a stick in the access hole to the termite mounds. The results showed that even though the animals somehow learned through several trial and errors to select the appropriate tool, they often used it in a wrong orientation, showing no solid understanding of how the tool should have been oriented and directed toward the mound’s opening.

The evidence concerning forms of natural tool use in macaque monkeys prompted the study of the underlying neural representation in the cerebral cortex. These studies were somehow rooted in the concept of “body scheme”, derived from neuropsychological studied of the last century. At the beginning of the twentieth century, in their exploration of how the human brain represents the body, Head and Holmes (1911) wrote: “It is to the existence of these “schemata” that we owe the power of projecting our recognition of posture, movement and locality beyond the limits of our own bodies to the end of some instrument held in the hand […]. Anything which participates in the conscious movement of our bodies is added to the model of ourselves and becomes part of these schemata”. The concept of body scheme remained for long time a sort of “happy metaphor disguising ignorance” (Mountcastle 1995) for the lack of solid knowledge on its neurophysiological substrates. Despite this, the body schemata concept influenced the neurological and psychological thinking of the last century and received some support from physiological studies in alert monkeys. Today, the classical concept of body scheme probably necessitates updating, so as to account for new theoretical arguments and experimental evidence, questioning the existence of a unique central representation of the peripersonal space (PPS), intended as a fixed geometrical construct within which we act around our body. Instead the PPS has been conceptualized as a set of continuously graded response fields, each reflecting the magnitude of physiological or behavioral measures related to the value of actions aiming to create or avoid contact between external objects and the body (de Vignemont and Iannetti 2015; Bufacchi and Iannetti 2018), in other words as a construct whose configuration is defined by the action affordances (Gibson 1979) available for behavior and eye-hand operations (Mascaro et al. 2003; Battaglia-Mayer and Caminiti 2018).

Probably inspired by Head and Holmes’ perspective, lriki et al. (1996) trained macaque monkeys to use a rake as a tool to retrieve distant objects of interest, such as a food pellet. In an intraparietal region overlapping parts of areas PE and PEip at the crown and dorsal bank of the IPS, they found bimodal neurons combining inputs from corresponding visual and somatosensory receptive fields (RFs) on the hand, a property regarded as the basis of a parietal mechanism responsible for encoding the hand schemata. These neurons were studied before and after the monkeys learned to use the rake. After tool use, the visual RF of neurons expanded, so as to include the far space reachable only with the rake. In the author’s interpretation, the rake was embodied in the animal’s body scheme, suggesting that an expansion of the subjective body image had occurred during tool use. This expansion of the vRF to the far region of space faded away when the hand and not the rake was used to retrieve the pellet, thus suggesting the existence of a fast form of tool-induced cross-modal plasticity, which inspired several behavioral studies in healthy human subject and in brain damaged patients (for a review see Maravita and Romano (2018) and the references therein). Similar results were observed in ventral premotor cortex after intense training in macaques using pliers (Umiltà et al. 2008). Different substrates were proposed to explain this phenomenon, such an extension of afferents from both the ventrolateral prefrontal areas and the temporo-parietal junction areas into the dorsal bank of the IPS (Hihara et al. 2006), increased level of neurotrophic factors and their receptors during tool use learning (Ishibashi et al. 2002a, b), increased gray-matter thickness (Quallo et al. 2009).

These studies made some intriguing predictions. First, the reported expansion of visual RF would predict a transformation of information from hand-centered to tool-centered coordinates, for which, however, quantitative evidence is still lacking. Second, the representation of the “peripersonal space” should also expand and the cross-modal interactions depending on the tool use re-encoded. Behavioral effects on body–space interactions dependent on tool use have been, indeed, described by Berti and Frassinetti (2000) in neglect patients, for whom the “far space” seems to be remapped as “near space” when bisecting lines using a tool. On a similar vein, Farnè et al. (2005, 2007) during tool use have documented an expansion of the peri-hand multisensory space that includes the distal part of the rake, and which depend on active tool use.

It is worth stressing that the very fast achievement of tool use may result from a bias in spatial attention toward the tool tip, rather than to its embodiment, and this hypothesis has generated a vivid debate, which still remains to be settled (see Maravita and Romano 2018). The study of tool selection remains ongoing, as a daily process dependent on the shape and size of the object to be retrieved. Tool selection provides grounds for future investigations in neurophysiology, as a special case of action selection.

It is worth noting that theories of tool embodiment do not account for the multiplicity of situations when tools are used on artifacts or materials of interest located at reach distance but potentially harmful, therefore, for danger or pain avoidance. An example from daily life is the use a wooden spoon to stir a hot soup. Similarly, chimpanzees often resort to making and using tools when the direct use of the hand could be potentially risky, as when searching for food in submerged areas. Under these circumstances, it has been hypothesized that separate non-isomorphic hand and tool representations are more advantageous and that chimpanzees use tools in a context-dependent fashion, as humans do (Povinelli et al. 2010). Flexible use of tools by chimpanzees has also been reported by (McGrew 1993).

The next step in monkey studies concerned the identification of the distributed system subserving tool use. Using positron emission tomography, Obayashi et al. (2001) confirmed that the cortical areas more activated during tool use were located in the contralateral IPS. Less consistently activated were a constellation of areas, such as motor cortex, supplementary motor area, precuneus, inferior temporal and cingulate cortex, and insula. Further activation was found in the basal ganglia and paramedian regions of the cerebellum. A subsequent study (Obayashi et al. 2003) showed the activation of a bilateral network including intraparietal, lateral prefrontal areas and deep cerebellar nuclei. This network was therefore hypothesized as a substrate of the inter-manual transfer of acquired tool use. Primary motor cortex might contribute to tool use (Quallo et al. 2012), thanks to its role in the fine control of digits movement, however it does not seem to play a causal role, since lesion of motor cortex does not result in tool apraxia.

The structural substrates of tool use in chimpanzees were conducted by analyzing the pattern of natural covariations in gray-matter across brains, through source-based morphometry (SBM) and by searching for heritable variations of its components by genetic analysis (Hopkins et al. 2019). Furthermore, the contribution of sex, age, as well as the phenotypic associations between gray-matter structural changes in different areas and skilled tool use were analyzed.

The results revealed several phenotypic correlations between tool use skills and gray-matter structure in superior temporal, parietal, and cerebellar cortex. For the first time, the heritability of some changes was suggested, since significant genetic correlations were found between tool use and the cuneus, the superior limb of the superior temporal sulcus (STS) and adjoining parietal cortex, the posterior sector of the STS, posterior cingulate cortex, visual cortex, and brainstem. At variance from previous studies in the literature, sex and age played a significant role. Sex influenced frontopolar and SPL areas probably related to affective, behavioral, and cognitive-motor functions. Indeed, although visuospatial functions are particularly sensitive to training and plastic responses (and hence to socio-cultural influences), males and females may have experienced different selective pressures for specific spatial capacities, during human evolution (Geary 2022). Age was studied trough body weight scores, and showed positive associations with prefrontal, premotor and lateral cerebellum, negative variations with superior frontal, SMA, and anterior temporal cortex. The conclusion of this study was that genetic mechanisms, yet to be discovered, might be responsible for the “[…] heritable link between variation in the capacity to use tools and variation in the morphology of the inferior and superior parietal lobe”.

Concerning allometric scaling and structural asymmetries in the IPL across macaques, chimpanzees and humans, Cheng et al. (2021) found a positive allometric pattern in both the right and left hemispheres, scaling across species. No connectional IPL asymmetries were found in macaques. Both chimpanzees and humans shared connectivity patterns characterized by leftward asymmetry in the anterior IPL areas, corresponding to the SMG, and rightward asymmetry in posterior IPL areas, concerning the AG. However, in humans, at variance from chimpanzees, the more diffuse leftward asymmetric networks included frontal, posterior parietal, and temporal areas, which might offer an anatomical substrate for the emergence of advanced tool use and language.

This association is of special interest, since in humans it might provide the neural substrate for representing the sematic knowledge about familiar skilled tools use. From an evolutionary perspective, the symmetric connectivity pattern between the IPL and the temporal areas shown in macaques probably evolved into an asymmetric connectional architecture in chimpanzees, achieving the highest expression in humans, thus shaping a trajectory potentially related to the emergence of complex tool use and language.

## Functional imaging and diffusion tractography studies of tool use in humans

Current understanding of the physiological substrates of tool use in humans stems from several fMRI and diffusion-weighted MRI tractography studies in healthy subjects and brain damaged patients. For the scope of this review, we will not discuss the extensive literature available on this subject, but only some studies that help in understanding how evolution has brought a separation of the cortical regions encoding object grasping from those subserving tool use in PPC.

fMRI experiments have studied the distributed system involved in tool use, starting from different questions and interests. Chao and Martin (2000), while studying category-related activities, predicted that only pictures of tools would activate cortical areas storing motor-related information. They found that viewing and naming pictures of tools activated in a selective fashion the left ventral premotor cortex and the SMG and assigned to these regions a role in tools recognition. Interestingly, Paleolithic tools trigger visual attention on functional tool parts and not on features associated with visual saliency (Silva-Gago et al. 2022). Attention is thus driven to different parts depending on whether the interaction is associated with ergonomic (hand-tool) or functional (tasks-related) expectation (Silva-Gago et al. 2021). This suggests that affordances and top-down processes are active during visual exploration of these tools, even in subjects with no previous archaeological knowledge.

Concerning behavioral phases of tool use, Johnson-Frey et al. (2005) distinguished regions involved in planning from those recruited during execution of tool use gestures. Planning activated a distributed system involving sectors of the superior temporal sulcus (STS), middle and superior temporal gyri, anterior and posterior SMG, angular gyrus (AG) and, in the frontal lobe, inferior frontal (IFC) and ventral premotor cortex (VPC), in addition to dlPFC. This activation pattern was independent from the arm involved. Gestures execution activated left IPL and inferior and middle frontal regions, beyond other areas classically involved in motor control and action sequences. Excluding contralateral sensory and motor cortex, this network was activated bilaterally, and this remained the main difference observed during execution vs planning. This study concluded that a left-hemisphere network is the neural substrate for the interaction between semantic and motor-related representations concerning skilled tool use.

By taking an evolutionary perspective, Peeters et al. (2009) compared the pattern of fMRI activation elicited by observation of common hand actions performed without and with simple tools in humans and macaque monkeys, including some animals trained to use a rake or pliers. In the first case, activation was observed in a bilateral distributed system including occipitoparietal, intraparietal and ventral premotor areas in both species. However, an additional tool-related activation was observed in the anterior SMG region in humans only. This area, regarded as essential to match tools observation and use and to establish causal relations between intention and consequences of tool use, was proposed as an emerging feature in evolution specific for tool use, and regardless of the type of tool used. This result was confirmed in a larger study in humans (Peeters et al. 2013) and discussed by Orban and Caruana (2014). It is worth stating, however, that an emerging new area in the anterior part of the IPL had not been recognized in studies based on diffusion tractography parcellation and comparison with resting state connectivity of IPL in humans vs. monkeys (Mars et al. 2011), as well in a study of IPL receptor architectonics (Caspers et al. 2013). According to both these studies, the aSMG region in humans (referred to as area PFop) would be homologous to the anterior IPL areas in monkeys. If so, this region should be considered as a specialization of an area and of connections already present in IPL of NHPs, rather than a new addition in evolution.

The potential consequences of the limited degree of freedom in tool use available to subjects during MRI scanning were considered in a study where subjects not only viewed images of tools and/or their use but were also presented with mock familiar 3-D tools that they were allowed to grasp (Stark and Zohary 2008). This revealed an activation along a functional gradient spanning the IPS, with the more caudal areas activated by the visual location of the tool, and the more medial and anterior areas by the identity of the acting hand.

Concerning tool use and affordances, Valyear et al. (2012) have stressed that familiar tools are visually encoded within the same areas related to skilled use based on previous experience, therefore where also action-relevant object properties, therefore affordances (Gibson 1979), are represented. This association might offer an evolutionary advantage.

By combining fMRI with pattern classification algorithms (Gallivan et al. 2013) showed that the regions activated by planned tool use, but silent during common hand actions, belong not only to the areas of the aSMG group, but also include the middle temporal gyrus (MTG), which is involved in storing semantic information about tools. This study showed that tool use is subserved by multiple frontoparietal distributed systems representing hand action, as well as hand- and tool-action planning, while parietal and occipitotemporal areas would encode hand action, body perception, and upcoming tool use. The authors concluded that “The highly specialized and hierarchical nature of this coding suggests that hand- and tool-related actions are represented separately at earlier levels of sensorimotor processing before becoming integrated in frontoparietal cortex”. Another recent imaging analysis also points to the distinction between the neural systems underlying haptically guided grasping and direct tool use (Styrkowiec et al. 2019).

The conclusion of most studies is that conceptual processing of tools would rely on connections linking frontal and parietal areas related to action knowledge, fusiform areas related to shape processing, posterior temporal, and intraparietal areas encoding abstract representations of tool knowledge. Interestingly, in subjects born without hands, due to upper limb dysplasia (Striem-Amit et al. 2017) and who use tools with their feet, the overlap between hand and tool representations in the occipitotemporal cortex is preserved, suggesting that the functional organization of this cortical region is largely innate. Changes in intrinsic functional connectivity have also been reported (Yoo et al. 2013), consisting in decreased coupling after practice between SMG and SPL, as well as between primary somatosensory cortex and cerebellum.

It is worth stressing that, due to technical difficulties and limitation inherent to the fMRI environment, so far neuroimaging studies in humans have never been performed during use of material tools like those of daily life and, even when material tools were used, the movement allowed were restricted to hand digits (Hermsdörfer et al. 2007). In this condition, a cortical network larger than that visualized during pantomimes of tool use emerged, together with significant difference in movement kinematics (Hermsdörfer et al. 2012). It is to be expected that future imaging studies, performed in more ecological conditions (therefore in subjects freely selecting and using common tools), together with improvements in fMRI technology, will change the current view on the neural basis of tool use. It remains to be determined to which extent this will happen.

Despite the persisting limitations of MRI-weighted diffusion tractography (Girard et al. 2020; Caminiti et al. 2021), several studies have attempted to unravel the cortical connectivity linking the areas activated by tool use. Ramayya et al. (2010) have identified three pathways, mostly in the left hemisphere. The first path, probably storing semantic information (tool identity, shape, function, and use) connects the posterior middle temporal gyrus (pMTG) with the aSMG (IPL-group I areas). A second system, linking the pSMG (IPL-group II/III areas) with the AG, might convey information about invariant features of tool gestures, such as planning, and mechanical sequences. A third system would convey to the motor output of the frontal lobe integrated semantic and non-spatial information, together with spatio-temporal representations, in other words, plans for tool use. This pathway connects the aSMG with a region of premotor cortex where tool use produces large fMRI activation peaks.

A different study addressed the causal role of different tracts by combining diffusion tractography, brain lesion, and behavioral performance (Bi et al. 2015). Three fronto-parietal and intrinsic frontal fiber tracts would be essential for tool use. These, together with five additional tracts linking frontal and temporo-parietal areas, would be crucial for conceptual understanding of tool use. All these systems travel in the superior longitudinal fascicle (SLF).

As a note of caution, it must be stressed that diffusion tractography visualizes streamlines and not structural cortico-cortical connections and does not provide information about their direction. Therefore, the direction of the information flow in the brain during tool use remains largely hypothetical.

## Neural basis of object construction

### Neuropsychological studies

As for tool use, the interest in the neural basis of object construction was prompted by the description of patients suffering from CA (constructional apraxia). This disorder is characterized by a disturbance “in formative activities such as assembling, building and drawing, in which the spatial form of the product proves to be unsuccessful, without there being an apraxia for single movements” (Wilson 1908). Critchley (1953) defined CA as a “difficulty in putting together one-dimensional units so as to form two-dimensional figures or patterns”. The difficulty to copy a visual model is essential for the definition of CA, which is diagnosed when a model object is presented to patients, and they are unable to produce a faithful copy of it (see Liepmann 1920; Strauss 1924; Morlaàs 1928; Kleist 1934; Goldenberg 2008, 2014; Gainotti and Trojano 2018). The copies produced are spatially disorganized; for instance, blocks are assembled into incorrect spatial relationships with respect to one another, so that the object’s structure is lost (Piercy et al. 1960; Benton and Fogel 1962; Benton 1967; Benson and Barton 1970). CA could be caused by a failure to effectively analyze the spatial structure of the model or by a difficulty in orchestrating the motor output to reproduce the model through appropriate action sequences (Mack and Levine 1981). The deficit is most severe after left PPC damage, though it can follow frontal cortex damage as well (Gainotti and Trojano 2018), as in patients with frontotemporal dementia, where defective figure copying is associated with spatial planning and working memory impairments and right dlPFC damage (Possin et al. 2011). Over the years, however, […] “the link between parietal lobe damage and CA have become more and more problematic with the development of more sophisticated neuropsychological models and methods of investigations” (for a discussion see Gainotti and Troiano 2018).

## Studies on construction behavior in non-human primates and humans

Constructional abilities in humans have mostly been studied during children development, by adopting a theoretical frame (Vereeken 1961; Stiles and Stern 2001) aimed at understanding how spatial knowledge emerges during ontogeny. This approach helped comparing constructive abilities in human vs non-human primates, such as chimpanzees, a species that diverged from the human clade about 6–7 million years ago, by searching for the presence of spontaneous and/or learned construction skills based on causal understanding of the goal.

It is beyond the scope of this review to offer a detailed discussion of these studies. In the wild, all chimpanzees build nests in a very short time, by adding on a foundation several leafy branches after bending them with hands or feet in a circle, so that the end-product is a roughly circular artifact around the animal, resulting from branch interweaving. Leaves and other soft materials are eventually added to make it more comfortable (Goodall 1962; Van Lawick-Goodall 1968; Baldwin et al. 1981; Potì et al. 2009). This example indicates that in the wild, chimpanzees’ spontaneous constructional skills rest on action sequences based on instinctive behavior, rather than on causal understanding of a project (Povinelli 2000).

**Fig. 5:**
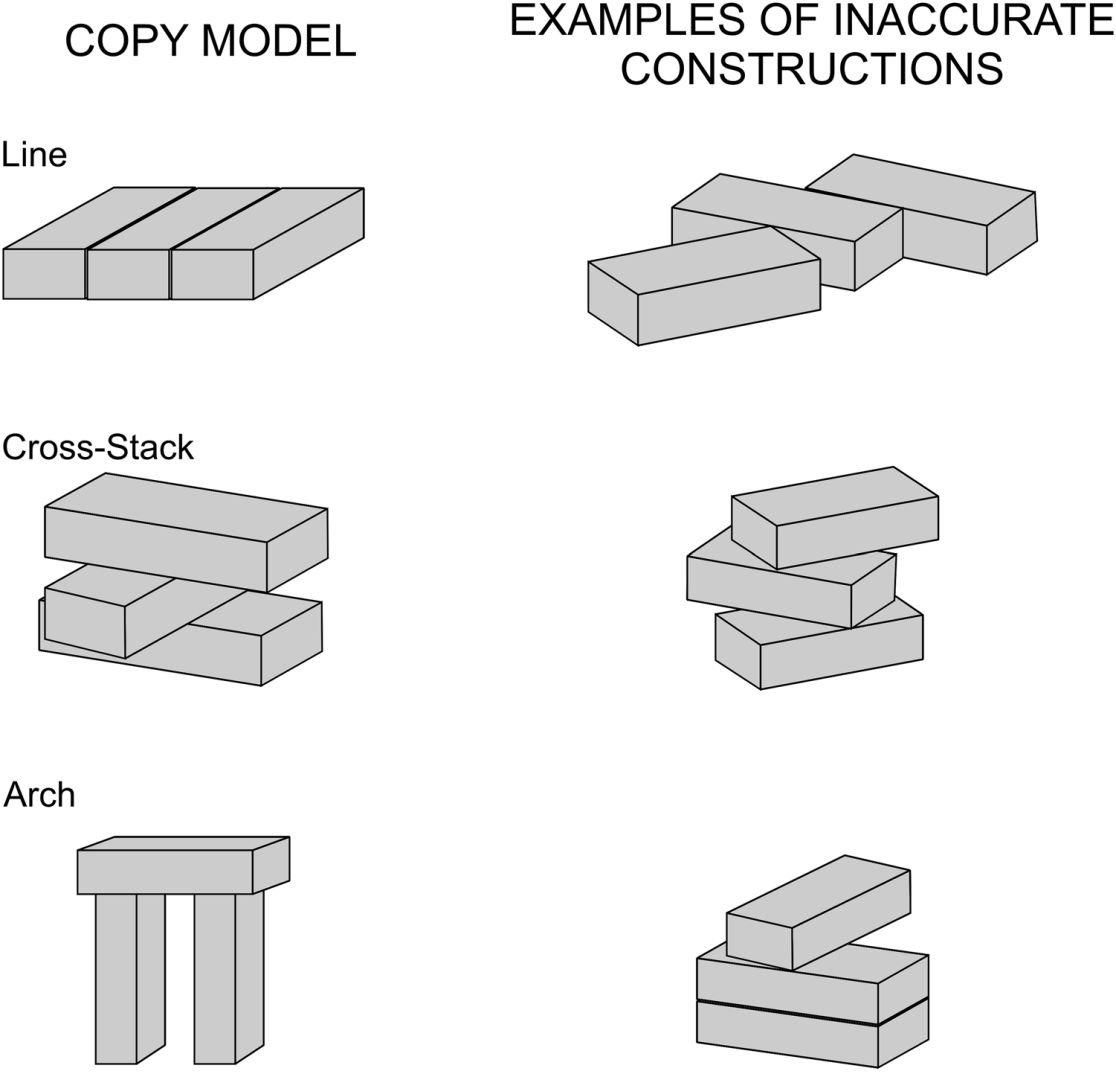
Copy-model construction task in chimpanzees. Left column: Copy-models formed by three horizontally aligned block of the same orientations (top); cross-stack formed by block crossing each other at 90°; Arch formed by two vertical blocks spanned by a third (horizontal) supported one. Right column: Chimpanzee’s inaccurate constructions, relative to the copy model. Line: blocks are placed in the floor in a different orientation; Cross-stack: blocks are placed one on top of the other with different angles, relative to the copy-model; Arch: proximity relations of block in the vertical dimensions are absent, and support relations are altered, therefore the copy does not reproduce the essential spatial relations of the Arch. (after Potì et al. 2009)

In the laboratory setting, chimpanzees showing construction abilities are only those raised in highly enriched natural environments since very early in life (Van Lawick-Goodall 1968). However, eliciting advanced constructions by them requires additional cues, such as showing a copy model (Potì et al. 2009). In their block modeling task, these authors used copy-model constructions made by three blocks (parallelepipeds) in three different configurations, Line, Cross-Stack, Arch (Fig. 5), of different types, spatial relations, and dimensions, all requiring similar configurational understanding. Constructing the first two implies only operating in one spatial relationship and in one-dimension (Horizontal, H), constructing Lines also implies next-to relations in the H dimension, Cross-Stacks imply a cross-and-support relation in the H dimension and, finally, building an Arch implies understanding of both next-to and support relations in the H and vertical (V) dimensions. The results of this study showed that after intensive training only adult chimpanzees can make some accurate constructions, starting with Cross-Stacks first, and blocks alignment in a Line after. At the age of 5, chimpanzees can manage cross and support relations. In children, this skill emerges at 5 months, and at about 9 months, objects are combined one into another to construct insertions (Langer 1980). Young chimps build mainly in the V dimension, but not in two dimensions, as children do from the age of 30 months. At this time, children construct along one dimension, and can master making cross-stacks and arches in one- or two-dimension at 42 months. Previous studies had shown that children spontaneously construct crossing relations in the vertical dimension (Vereeken 1961) at about 30–36 months, and spatial grouping relations are first made along one-dimension before two-dimensions (Guanella 1934; Stiles-Davis 1988). Accuracy in children for constructional praxis increases very rapidly over time and is fully mature at the age of 7 years.

In summary, adult chimps rarely make stable bi-dimensional constructions, cannot build simple objects requiring the analysis of simultaneous spatial relationships, never coordinate in systematic fashion multiple relations between separate constructions or combine two constructions into one. This is considered as a crucial limit of their constructional skills, since it prevents building more complex architectures, such as enclosed 3-D spaces (Potì and Langer 2001; Potì 2005; Potì et al. 2009), as children do at the age 3 years (Guanella 1934). Finally, adult chimps never develop the ability to construct nonfunctional symmetrical spatial relationships (Potì and Langer 2001), such as placing objects next to each other. Overall, their constructions at the best remain like those of 2-year-old children, and similar in this to brain damaged children (Stiles-Davis et al. 1985).

Although these studies showed some construction skills never described before in chimpanzees, as a note of caution Potì et al. (2009) recognized that a crucial limitation of studies on chimpanzees’ constructive abilities in laboratory settings is that the protocols adopted allowed the animal to observe the construction of the copy-model by the experimenter.

Beyond child development, to our knowledge there is no detailed quantitative analysis of human behavior, especially on eye-hand coordination, during construction tasks under naturalistic conditions. In Ballard’s et al. (1992) copy task, a gold standard for such studies, subjects observed on a computer screen a copy model consisting of colored blocks, and were asked to copy it by selecting, with a computer mouse, the appropriate blocks from a distant source area and bring them to be assembled within a near work-space area. Initially, the hand moved to the source area and stayed there, while the eye first gazed to the copy model, then onto a block located in the source area, as if to guide the upcoming hand pick-up action. Then the eye returned to the model, the hand moved to the block drop-off location, while in the meantime the eye had returned to the drop-off area to guide the block release by the hand. Thus, a block was generally fixated just before its use and the saccadic system prompted key changes just when required by the status of the hand. This continuous access to sensory information during the copy-task allows the use of sequences of primitives, termed *deictic* by (Agre and Chapman 1987), which implicitly predict the content of the next one in the sequential order of behavior (Lashley, 1951). In this, as in other tasks, such as car driving (Land and Lee 1994) and tea-making (Land et al. 1999), the brain seems to use a “do it where you look” strategy (Ballard et al. 1992), thanks to which objects crucial to the control system are placed at the center of vision. It is therefore of interest that in patients with CA due to right brain damage, a significant deficit was found in remapping spatial locations across saccades, especially when the first saccade was directed to the right, such that eye movements resulted in loss of remembered spatial information from previous fixation periods (Russell et al. 2010). These results suggest that a defective remapping of visual information across saccadic eye movements contributes to CA, in line with the observation on eye behavior during Ballard’s copy task.

## Neurophysiological studies on object construction

Unfortunately, only few neurophysiological studies relevant to understanding constructional praxis are available in NHPs. These studies assumed that there exist at least two fundamental processes underlying object construction, namely the spatial cognitive analysis of object structure, based on a copy model, and the specification of the orderly sequence of eye and hand movement necessary to assemble an object from its elementary components, in other words, the serial order of behavior (Lashley 1951).

**Fig. 6:**
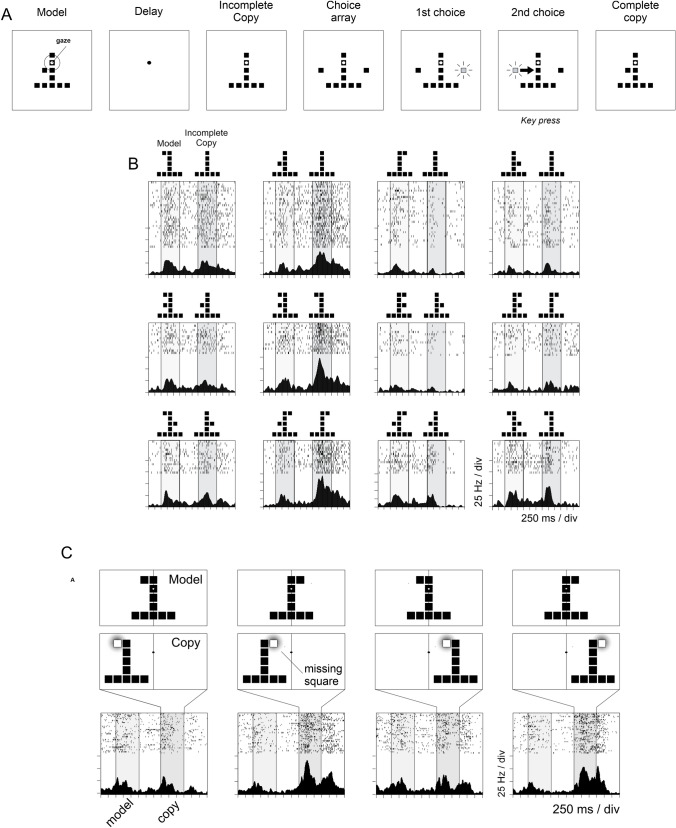
Neural activity recorded in inferior parietal area 7a during an object construction task (“copy task”) where monkeys were required to identify the missing component of a copy-model stored in the object construction task (left to right). **A** A visual target was first presented in the centre of a screen and the monkeys kept fixation on it throughout the trial (‘gaze’). Then, a copy-model was presented (‘Model’) made by a varying configuration of square elements. After a variable delay, another copy object was presented, identical to the previous one but with a single missing square. The monkeys had to replace the missing square, so as to restore the configuration of the copy-model model. For this, it had to select from a Choice array one of the two presented squares that brightened one at a time in random order, by depressing a single response key. If the monkey pressed the key at the correct time, the bright square was added automatically to the object. **B** Activity of a single parietal cell during the task. Raster plots show neural activity in different trials for one of the possible combinations of model and copy objects. Vertical lines in each raster delineate time epochs when the model and the copy objects were visible. This cell was mainly activated during the copy epoch, on trials in which the missing square was on the lower left position in the object, which was the position for which the cell activity was highest (preferred position: second column from left). Identical copy objects were displayed at the same position in the display in all the rasters across the top row, but activity during the copy period clearly varied depending on the location of the missing square, therefore it did not encode the form or position of the copy object. Similarly, the neural signal did not code the direction of the motor response, which was invariant over trials. Therefore, neural activity reflected cognitive variables underlying the value of the spatial position of the missing square to the solution of the problem posed by the construction task (Modified from Chafee et al. 2005). **C** Neural activity in area 7a represents object-centered position during the construction task. Activity of a single neuron in area 7a modulated during the copy period when the missing square was from the preferred *right* side of the copy object (second and fourth columns from left), and not when it was missing from the non-preferred *left* side of the copy object (first and third columns). The neural activity remained strong irrespective of whether the missing critical square and copy object were in the left (second column) or the right visual hemifield (fourth column). This suggest that neural activity encoded the horizontal position of the missing square, right or left, relative to the object midline, therefore in object-centered coordinates, regardless of the gaze fixation target, therefore of the viewer-centered reference frame

Under these perspectives, macaque monkeys were trained in a task requiring the analysis of object structure (Chafee et al. 2005, 2007; Crowe et al. 2008) and, after training, cell activity was recorded in IPL (area 7a, PG) while they performed the task. A *model* object was first presented, consisting of varying configurations of square components (Fig. 6A ‘Model’). After a delay time, an uncomplete *copy* of the same *model* was presented, differing in a single component (one square) that was missing (Fig. 6A ‘Uncomplete Copy’). Then a choice array was shown with two squares flanking the right and left of the copy model, brightening one at the time in a random fashion. To restore the complete copy, the animal had to select the missing component between two possible choices presented sequentially (Fig. 6A ‘1st choice’, ‘2nd choice’), by pressing a response key when the correct choice was presented. The sequential choice behavioral report facilitated the identification of neural activities related to the analysis of object structure, rather than to the direction of forthcoming hand movement. Neural activity varied depending on the location of the missing component even when the form and position of the copy object remained constant (Fig. 6C), thus excluding influences dependent of the spatial characteristics of the visual input. Furthermore, the spatial information encoded by neural activity was not correlated with the direction of the upcoming motor action, which remained invariant across trials. This suggests that neural activity reflected the spatial cognitive process analyzing the object structure necessary to direct the replacement of the missing component. Separate populations of neurons encoded the position of the missing components in viewer-centered or in object-centered spatial coordinates (Chafee et al. 2007). One neural ensemble represented the position in object-centered coordinates, since neural activity varied depending on the left or right position of it, relative to the midline of the object. Another population instead encoded the missing component in viewer-centered coordinates, since cell activity varied depending on whether it was located to the left or right of the gaze fixation target, that is at the midline of the viewer-centered reference frame. A decoding analysis revealed that variations in viewer-centered coordinates led in time and predicted variations in the object-centered frames within a trial (Crowe et al. 2008). Therefore, parietal area 7a encoded position provided by the visual input initially in retino-centric coordinates and this information was then transformed into a spatial coding of position in object-centered coordinates, so as to direct the construction task.

These elegant studies were designed to simulate constructional praxis in humans and provided compelling evidence that parietal neurons encode a spatial cognitive process that analyzes object structure upstream of motor processing. In fact, the construction task did not require monkeys to assembly objects, therefore it remains to be determined how signals reflecting object structure ultimately shape motor intention and outflow to direct the manual construction of complex material artifacts.

**Fig. 7:**
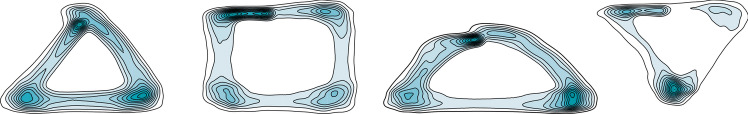
Density plots of trajectories obtained by estimating the positions occupied by a visual cursor moved by the monkey’s hand acting on a joystick, sampled at each position along the trajectory. Higher density indicates slower movement and more trajectories passing through that location (modified from Averbeck et al. 2009)

Although the final answer to this question can only be achieved by recording neural activity from the human brain during object construction (given that only humans display elaborated constructional abilities), a further step toward understanding the neural bases of the sequential order of actions required for such task consisted in recording and comparing cell activity in dlPFC (Averbeck et al. 2002) and SPL (Averbeck et al. 2009) in a copy task, inspired by the concept of serial order of behavior (Lashley, 1951). Macaque monkeys were trained to copy geometrical shapes such as triangles, squares, trapezoids, and inverted triangles (Fig. 7) as sequences of movement segments, and these were associated to the activity of single cells and of populations of neurons recorded simultaneously. It was found that shape and segment serial position (cognitive factors), as well as hand speed, position, and direction of the segment (motor factors) influenced the activity of individual cells more in parietal than in dlPFC. However, consistent information about hand velocity was also found in dlPFC, although this information was more accurately represented in parietal cell assemblies and as a function of their size. Concerning timing of activation, an equal amount of parietal activity led and lagged hand velocity, while in dlPFC neural activity lagged hand velocity, suggestive of the existence of a predominantly sensory representation in the latter vs. a more balanced sensorimotor encoding in the former. Figure shape was better encoded in parietal than in dlPFC, with about 40–50% neurons modulated by the shape being drawn. In both areas, serial position was encoded together with shape, direction, and segment length, revealing the combinatorial power of neural activity.

From a behavioral point of view, errors were more common in the middle of the drawing sequence, rather than at its beginning and end. Interestingly, neural activity on errors trials did not generally encode the segment being drawn, whose neural representation was therefore classified as weak. On error trials, subsequent segments were more likely encoded, with individual elements of the sequence represented in a parallel fashion within the neural ensemble before the sequence execution.

These results suggest that correct drawing of figures consists of a motor sequence made of individual movement segments of given direction and length, drawn in a precise serial order.

Although they have been discussed in relation of ideomotor and ideational apraxia and agraphia, these data are also relevant to CA, where the serial order of eye and hand movement appear to be crucial for successful construction of complex structures based on copy models. In fact, after Strauss (1924), Kleist (1934) and Critchley (1953) perspectives on CA, the main test requested to patients to diagnose this disorder consists in copying figures composed of multiple segments arranged in given spatial relations, rather than drawing or tracing single lines (for a discussion, see Raimo et al. 2021), or constructing complex objects.

Behavioral neurophysiological studies, such as those illustrated above, then represent a crucial step toward understanding the neural substrates object construction and an example for future analysis in humans using tools to construct complex artifacts. Exciting works on this issue is warranted for future generations.

## Conclusions

Two million years after the emergence of a material culture, the human genus has developed a very advanced and elaborated ability to use tools and construct complex artifacts, and to integrate these peripheral elements as active components of cognitive processes. This capacity to include tools as prosthetic extension of the body can be even detected experimentally, and largely involves the activation of the posterior parietal cortex (Miller et al. 2018, 2019). Behavioral neurophysiology, neuroanatomy and neuroimaging have highlighted the importance of the parietal lobes in the management of such prosthetic capacity by showing how tools can be used and “incorporated” in the body scheme, thanks to the neural operations and interplay within a distributed system including parietal, temporal, and frontal areas. Behavioral neuroscience has shown how construction of complex manufactures rests on the visuo-spatial analysis of a physical or mental copy-model and on the serial order of eye-hand coordinated action, as when assembling a lego from blocks, creating a mosaic from its component tessera, or copying geometrical figures. Modern achievements on the neural substrates of tool use and object construction were inspired by neuropsychological studies on their disorders, i.e., tool apraxia and constructional apraxia, first described at the beginning of the last century in patients with brain damage affecting mostly the parietal cortex but surprisingly devoid of primary sensory and motor disorders. Tool and constructional apraxia can now be regarded as disturbances of the cognitive aspects of motor control. Studies of comparative behavior across species have shown that, at variance for macaques and chimpanzees, which can use tools or build nest by associative learning or instinct, only humans possess causal understanding of the final goal underlying such complex tasks, as well as of the way to achieve it.

At the same time, theories on extended cognition have supplied a theoretical frame to interpret “mind” as a process generated by the interaction between brain, body, and culture. Including evolutionary anthropology and the study of extinct human species can, in this sense, add a relevant contribution to this perspective. Paleoneurology has demonstrated that modern humans—and, to a lesser extent, Neandertals—experienced a morphological expansion of the parietal regions (Bruner 2018). Further studies on fossils can manage to increase the spatial resolution of such evidence, detecting specific local variation of the cerebral surface (Pereira-Pedro et al. 2020). Neuroarchaeology has already begun to focus on neurofunctional responses to hand–tool interaction, stressing further the importance of a human-specific parietal circuitry when dealing with technological integration (Stout and Hecht 2017). Cognitive archaeology is investigating the visual exploration and haptic feedback to body-tool stimulation (Fedato et al. 2019; Silva-Gago et al. 2021). All these fields and research areas are prompting a renewed interest toward the parietal cortex, visuospatial integration, and body cognition in evolutionary anthropology, bridging prehistory and cognitive sciences (Bruner et al. 2018c).

One of the main issues of debate and sources of disagreement in almost two centuries of modern evolutionary theories concerns whether the differences between humans and other species are a matter of degree and continuity, or due to specific features and discontinuity. Assuming that the most probable situation deals with an admixture of both quantitative and qualitative changes, we must recognize that the results is, undoubtedly, astonishing. Even if only by a matter of grade, our cognitive capacities are unique in many aspects. We have at present many cues suggesting that such uniqueness is also due to a specialized parietal cortex, and to its involvement in interfacing our own body with social, symbolic, and technological extensions.

## Data Availability

Enquiries about data availability should be directed to the authors.

## References

[CR1] Agre P, Chapman D (1987). Pengi: an implementation of a theory of activity.

[CR2] Amunts K, Zilles K (2015). Architectonic mapping of the human brain beyond Brodmann. Neuron.

[CR3] Avants BB, Schoenemann PT, Gee JC (2006). Lagrangian frame diffeomorphic image registration: morphometric comparison of human and chimpanzee cortex. Med Image Anal.

[CR4] Averbeck BB, Chafee MV, Crowe DA, Georgopoulos AP (2002). Parallel processing of serial movements in prefrontal cortex. Proc Natl Acad Sci.

[CR5] Averbeck BB, Crowe DA, Chafee MV, Georgopoulos AP (2009). Differential contribution of superior parietal and dorsal–lateral prefrontal cortices in copying. Cortex.

[CR6] Baldwin PJ, Pí JS, McGrew WC, Tutin CEG (1981). Comparisons of nests made by different populations of chimpanzees (Pan troglodytes). Primates.

[CR7] Ballard DH, Hayhoe MM, Li F, Whitehead SD (1992). Hand-eye coordination during sequential tasks. Philos Trans R Soc Lond B.

[CR8] Battaglia-Mayer A (2001). Eye-hand coordination during reaching. II. An analysis of the relationships between visuomanual signals in parietal cortex and parieto-frontal association projections. Cereb Cortex.

[CR9] Battaglia-Mayer A, Caminiti R (2018). Parieto-frontal networks for eye–hand coordination and movements. Handbook of clinical neurology.

[CR10] Battaglia-Mayer A, Caminiti R (2019). Corticocortical systems underlying high-order motor control. J Neurosci.

[CR11] Battaglia-Mayer A, Ferraina S, Mitsuda T (2000). Early coding of reaching in the parietooccipital cortex. J Neurophysiol.

[CR12] Benson DF, Barton MI (1970). Disturbances in constructional ability. Cortex.

[CR13] Benton AL (1967). Constructional apraxia and the minor hemisphere. Confin Neurol.

[CR14] Benton AL, Fogel ML (1962). Three-dimensional constructional praxis: a clinical test. Arch Neurol.

[CR15] Bergson H (1907). L’évolution Créatrice.

[CR16] Berlucchi G, Vallar G (2018). The history of the neurophysiology and neurology of the parietal lobe. Handbook of clinical neurology.

[CR17] Berti A, Frassinetti F (2000). When far becomes near: remapping of space by tool use. J Cogn Neurosci.

[CR18] Bi Y, Han Z, Zhong S (2015). The white matter structural network underlying human tool use and tool understanding. J Neurosci.

[CR19] Binder JR, Desai RH, Graves WW, Conant LL (2009). Where is the semantic system? A critical review and meta-analysis of 120 functional neuroimaging studies. Cereb Cortex.

[CR20] Borra E, Gerbella M, Rozzi S, Luppino G (2017). The macaque lateral grasping network: a neural substrate for generating purposeful hand actions. Neurosci Biobehav Rev.

[CR21] Boyd R (2018). A different kind of animal: how culture transformed our species.

[CR22] Bruner E (2004). Geometric morphometrics and paleoneurology: brain shape evolution in the genus Homo. J Hum Evol.

[CR23] Bruner E, Bruner E (2015). Functional craniology and brain evolution. Human paleoneurology.

[CR24] Bruner E, Kaas J (2017). The fossil evidence of human brain evolution. Evolution of nervous systems.

[CR25] Bruner E (2018). Human paleoneurology and the evolution of the parietal cortex. Brain Behav Evol.

[CR26] Bruner E (2019). Human paleoneurology: shaping cortical evolution in fossil hominids. J Comp Neurol.

[CR27] Bruner E (2021). Evolving human brains: paleoneurology and the fate of middle pleistocene. J Archaeol Method Theory.

[CR28] Bruner E, Gleeson BT (2019). Body cognition and self-domestication in human evolution. Front Psychol.

[CR29] Bruner E, Holloway RL (2010). A bivariate approach to the widening of the frontal lobes in the genus Homo. J Hum Evol.

[CR30] Bruner E, Iriki A (2016). Extending mind, visuospatial integration, and the evolution of the parietal lobes in the human genus. Quatern Int.

[CR31] Bruner E, Lozano M (2014). Extended mind and visuo-spatial integration: three hands for the Neandertal lineage. J Anthropol Sci.

[CR32] Bruner E, Lozano M (2015). Three hands: one year later. J Anthropol Sci.

[CR33] Bruner E, Pearson O (2013). Neurocranial evolution in modern humans: the case of Jebel Irhoud 1. As.

[CR34] Bruner E, Manzi G, Arsuaga JL (2003). Encephalization and allometric trajectories in the genus Homo: evidence from the Neandertal and modern lineages. Proc Natl Acad Sci.

[CR35] Bruner E, De La Cuétara JM, Holloway R (2011). A bivariate approach to the variation of the parietal curvature in the genus homo. Anat Rec.

[CR2001] Bruner E, de la Cuétara JM, Masters M, Amano H, Ogihara N (2014). Functional craniology and brain evolution: from paleontology to biomedicine. Front Neuroanat.

[CR36] Bruner E, Amano H, de la Cuétara JM, Ogihara N (2015). The brain and the braincase: a spatial analysis on the midsagittal profile in adult humans. J Anat.

[CR37] Bruner E, Preuss TM, Chen X, Rilling JK (2017). Evidence for expansion of the precuneus in human evolution. Brain Struct Funct.

[CR38] Bruner E, Fedato A, Silva-Gago M (2018). Cognitive archeology, body cognition, and hand–tool interaction. Progress in brain research.

[CR39] Bruner E, Fedato A, Silva-Gago M, Hodgson T (2018). Visuospatial integration and hand-tool interaction in cognitive archaeology. Processes of visuospatial attention and working memory.

[CR40] Bruner E, Spinapolice E, Burke A, Overmann KA, Di Paolo LD, Di Vincenzo F, De Petrillo F (2018). Visuospatial integration: paleoanthropological and archaeological perspectives. Evolution of primate social cognition.

[CR41] Bufacchi RJ, Iannetti GD (2018). An action field theory of peripersonal space. Trends Cogn Sci.

[CR42] Buxbaum LJ, Kyle K, Grossman M, Coslett B (2007). Left inferior parietal representations for skilled hand-object interactions: evidence from stroke and corticobasal degeneration. Cortex.

[CR43] Buxbaum LJ, Shapiro AD, Coslett HB (2014). Critical brain regions for tool-related and imitative actions: a componential analysis. Brain.

[CR44] Bzdok D, Hartwigsen G, Reid A (2016). Left inferior parietal lobe engagement in social cognition and language. Neurosci Biobehav Rev.

[CR45] Caminiti R, Innocenti GM, Battaglia-Mayer A (2015). Organization and evolution of parieto-frontal processing streams in macaque monkeys and humans. Neurosci Biobehav Rev.

[CR46] Caminiti R, Borra E, Visco-Comandini F (2017). Computational architecture of the parieto-frontal network underlying cognitive-motor control in monkeys. eNeuro.

[CR47] Caminiti R, Girard G, Battaglia-Mayer A (2021). The complex hodological architecture of the macaque dorsal intraparietal areas as emerging from neural tracers and DW-MRI tractography. eNeuro.

[CR48] Caspers S, Zilles K (2018). Microarchitecture and connectivity of the parietal lobe. Handbook of clinical neurology.

[CR49] Caspers S, Schleicher A, Bacha-Trams M (2013). Organization of the human inferior parietal lobule based on receptor architectonics. Cereb Cortex.

[CR50] Chafee MV, Crowe DA, Averbeck BB, Georgopoulos AP (2005). Neural correlates of spatial judgement during object construction in parietal cortex. Cereb Cortex.

[CR51] Chafee MV, Averbeck BB, Crowe DA (2007). Representing spatial relationships in posterior parietal cortex: single neurons code object-referenced position. Cereb Cortex.

[CR52] Chao LL, Martin A (2000). Representation of manipulable man-made objects in the dorsal stream. Neuroimage.

[CR53] Cheng L, Zhang Y, Li G (2021). Connectional asymmetry of the inferior parietal lobule shapes hemispheric specialization in humans, chimpanzees, and rhesus macaques. Elife.

[CR55] Collias NE (1964). The evolution of nests and of nests-building in birds. Am Zool.

[CR56] Coolidge FL, Overmann KA (2012). Numerosity, abstraction, and the emergence of symbolic thinking. Curr Anthropol.

[CR57] Coolidge FL, Wynn T (2005). Working memory, its executive functions, and the emergence of modern thinking. CAJ.

[CR58] Coolidge FL, Overmann KA, Wynn T (2011). Recursion: what is it, who has it, and how did it evolve?. Wires Cogn Sci.

[CR59] Coolidge FL, Wynn T, Overmann KA, Hicks JM, Bruner E (2015). Cognitive archaeology and the cognitive sciences. Human paleoneurology.

[CR60] Critchley M (1953). The parietal lobes.

[CR61] Crowe DA, Averbeck BB, Chafee MV (2008). Neural ensemble decoding reveals a correlate of viewer- to object-centered spatial transformation in monkey parietal cortex. J Neurosci.

[CR200] Donkervoort M, Dekker J, van den Ende E, Stehmann-Saris JC, Deelman BG (2000). Prevalence of apraxia among patients with a first left hemisphere stroke in rehabilitation centres and nursing homes. Clin Rehabil.

[CR62] De Renzi E, Lucchelli F (1988). Ideational apraxia. Brain.

[CR63] de Vignemont F, Iannetti GD (2015). How many peripersonal spaces?. Neuropsychologia.

[CR64] Farnè A, Iriki A, Làdavas E (2005). Shaping multisensory action–space with tools: evidence from patients with cross-modal extinction. Neuropsychologia.

[CR65] Farnè A, Serino A, Làdavas E (2007). Dynamic size-change of peri-hand space following tool-use: determinants and spatial characteristics revealed through cross-modal extinction. Cortex.

[CR66] Fedato A, Silva-Gago M, Terradillos-Bernal M (2019). Electrodermal activity during Lower Paleolithic stone tool handling. Am J Hum Biol.

[CR67] Fedato A, Silva-Gago M, Terradillos-Bernal M (2020). Hand morphometrics, electrodermal activity, and stone tools haptic perception. Am J Hum Biol.

[CR68] Ferrari-Toniolo S, Visco-Comandini F, Papazachariadis O (2015). Posterior parietal cortex encoding of dynamic hand force underlying hand-object interaction. J Neurosci.

[CR69] Gainotti G, Trojano L (2018). Constructional apraxia. Handbook of clinical neurology.

[CR70] Gallivan JP, McLean DA, Valyear KF, Culham JC (2013). Decoding the neural mechanisms of human tool use. Elife.

[CR71] Geary DC (2022). Spatial ability as a distinct domain of human cognition: an evolutionary perspective. Intelligence.

[CR72] Gibson JJ (1979). The ecological approach to visual perception: classic edition.

[CR73] Girard G, Caminiti R, Battaglia-Mayer A (2020). On the cortical connectivity in the macaque brain: a comparison of diffusion tractography and histological tracing data. Neuroimage.

[CR74] Goldenberg G (2008). Apraxia. Handbook of clinical neurology.

[CR75] Goldenberg G (2014). Apraxia—the cognitive side of motor control. Cortex.

[CR76] Goldenberg G, Hagmann S (1998). Tool use and mechanical problem solving in apraxia. Neuropsychologia.

[CR77] Goldenberg G, Spatt J (2009). The neural basis of tool use. Brain.

[CR78] Goldring AB, Krubitzer LA (2020). Evolution of Parietal Cortex in Mammals: From Manipulation to Tool Use. Evolutionary Neuroscience.

[CR79] Goodall JM (1962). Nest building behavior in the free ranging chimpanzee. Ann N Y Acad Sci.

[CR80] Goodall J (1986). The chimpanzees of Gombe: patterns of behavior.

[CR81] Gould JL, Gould CG (2012). Animal architects: building and the evolution of intelligence.

[CR82] Graves WW, Desai R, Humphries C (2010). Neural systems for reading aloud: a multiparametric approach. Cereb Cortex.

[CR83] Grefkes C, Fink GR (2005). The functional organization of the intraparietal sulcus in humans and monkeys. J Anat.

[CR84] Guanella FM (1934). Block building activities of young children. Arch Psychol.

[CR85] Gumert MD, Hoong LK, Malaivijitnond S (2011). Sex differences in the stone tool-use behavior of a wild population of burmese long-tailed macaques (Macaca fascicularis aurea). Am J Primatol.

[CR86] Hansell M (2000). Bird Nests and construction behaviour.

[CR87] Hansell MH (2005). Animal architecture.

[CR88] Hansell M, Ruxton G (2008). Setting tool use within the context of animal construction behaviour. Trends Ecol Evol.

[CR89] Head H, Holmes G (1911). Sensory disturbances from cerebral lesions. Brain.

[CR90] Hécaen H, Assal G (1970). A comparison of constructive deficits following right and left hemispheric lesions. Neuropsychologia.

[CR91] Hermsdörfer J, Terlinden G, Mühlau M (2007). Neural representations of pantomimed and actual tool use: evidence from an event-related fMRI study. Neuroimage.

[CR92] Hermsdörfer J, Li Y, Randerath J (2012). Tool use without a tool: kinematic characteristics of pantomiming as compared to actual use and the effect of brain damage. Exp Brain Res.

[CR93] Hihara S, Notoya T, Tanaka M (2006). Extension of corticocortical afferents into the anterior bank of the intraparietal sulcus by tool-use training in adult monkeys. Neuropsychologia.

[CR94] Hirsch ES (1996). The block book.

[CR95] Holloway RL (1981). Exploring the dorsal surface of hominoid brain endocasts by stereoplotter and discriminant analysis. Philos Trans R Soc Lond B.

[CR96] Holloway RL, Broadfield DC, Yuan MS (2004). The human fossil record, vol. 3, brain endocasts, the paleoneurological evidence.

[CR97] Hopkins WD, Latzman RD, Mareno MC (2019). Heritability of gray matter structural covariation and tool use skills in Chimpanzees (*Pan troglodytes*): a source-based morphometry and quantitative genetic analysis. Cereb Cortex.

[CR98] Iriki A, Taoka M (2012). Triadic (ecological, neural, cognitive) niche construction: a scenario of human brain evolution extrapolating tool use and language from the control of reaching actions. Philos Trans R Soc B.

[CR99] Iriki A, Tanaka M, Iwamura Y (1996). Coding of modified body schema during tool use by macaque postcentral neurones. NeuroReport.

[CR100] Ishibashi H, Hihara S, Takahashi M (2002). Tool-use learning induces BDNF expression in a selective portion of monkey anterior parietal cortex. Mol Brain Res.

[CR101] Ishibashi H, Hihara S, Takahashi M (2002). Tool-use learning selectively induces expression of brain-derived neurotrophic factor, its receptor trkB, and neurotrophin 3 in the intraparietal multisensorycortex of monkeys. Cogn Brain Res.

[CR102] Jeannerod M, Arbib MA, Rizzolatti G, Sakata H (1995). Grasping objects: the cortical mechanisms of visuomotor transformation. Trends Neurosci.

[CR103] Johnson-Frey SH, Newman-Norlund R, Grafton ST (2005). A distributed left hemisphere network active during planning of everyday tool use skills. Cereb Cortex.

[CR104] Kaas JH, Qi H-X, Stepniewska I (2018). The evolution of parietal cortex in primates. Handbook of clinical neurology.

[CR105] Krakauer JW, Carmichael ST (2017). Broken movement: the neurobiology of motor recovery after stroke.

[CR106] Land MF, Lee DN (1994). Where we look when we steer. Nature.

[CR107] Land M, Mennie N, Rusted J (1999). The roles of vision and eye movements in the control of activities of daily living. Perception.

[CR108] Langer J (1980). The origins of logic: six to twelve months.

[CR109] Lashley KS (1951) The problem of serial order in behavior. In: Jeffress LA (ed) Cerebral mechanisms in behavior. Wiley, New York

[CR110] Liepmann H (1905). Die linke Hemisph€are und das Handeln. Munch Med Wochenschr.

[CR111] Liepmann H (1920). Apraxie. Ergb Gesamte Med.

[CR112] Mack JL, Levine RN (1981). The basis of visual constructional disability in patients with unilateral cerebral lesions. Cortex.

[CR113] Mainwaring MC, Hartley IR, Lambrechts MM, Deeming DC (2014). The design and function of birds’ nests. Ecol Evol.

[CR114] Malafouris L (2010). The brain–artefact interface (BAI): a challenge for archaeology and cultural neuroscience. Soc Cognit Affect Neurosci.

[CR115] Maravita A, Romano D (2018). The parietal lobe and tool use. Handbook of clinical neurology.

[CR116] Mars RB, Jbabdi S, Sallet J (2011). Diffusion-weighted imaging tractography-based parcellation of the human parietal cortex and comparison with human and macaque resting-state functional connectivity. J Neurosci.

[CR117] Mascaro M, Battaglia-Mayer A, Nasi L (2003). The eye and the hand: neural mechanisms and network models for oculomanual coordination in parietal cortex. Cereb Cortex.

[CR118] Mayer-Gross W (1935). Some observations on Apraxia: (section of neurology). Proc R Soc Med.

[CR119] McGrew W, Gibson K, Ingold T (1993). The intelligent use of tools: twenty propositions. Tools, language and cognition in human evolution.

[CR120] Miller LE, Montroni L, Koun E (2018). Sensing with tools extends somatosensory processing beyond the body. Nature.

[CR121] Miller LE, Fabio C, Ravenda V (2019). Somatosensory cortex efficiently processes touch located beyond the body. Curr Biol.

[CR122] Morlaàs J (1928). Contribution a’ l’étude de l’apraxie.

[CR123] Mountcastle VB (1995). The parietal system and some higher brain functions. Cereb Cortex.

[CR124] Neubauer S, Hublin J-J, Gunz P (2018). The evolution of modern human brain shape. Sci Adv.

[CR125] Obayashi S, Suhara T, Kawabe K (2001). Functional brain mapping of monkey tool use. Neuroimage.

[CR126] Obayashi S, Suhara T, Kawabe K (2003). Fronto-parieto-cerebellar interaction associated with intermanual transfer of monkey tool-use learning. Neurosci Lett.

[CR127] Orban GA, Caruana F (2014). The neural basis of human tool use. Front Psychol.

[CR128] Osiurak F, Jarry C, Le Gall D (2010). Grasping the affordances, understanding the reasoning: toward a dialectical theory of human tool use. Psychol Rev.

[CR129] Palomero-Gallagher N, Zilles K (2018). Cyto- and receptor architectonic mapping of the human brain. Handbook of clinical neurology.

[CR130] Passingham RE, Smaers JB (2014). Is the prefrontal cortex especially enlarged in the human brain? Allometric relations and remapping factors. Brain Behav Evol.

[CR131] Peeters R, Simone L, Nelissen K (2009). The representation of tool use in humans and monkeys: common and uniquely human features. J Neurosci.

[CR132] Peeters RR, Rizzolatti G, Orban GA (2013). Functional properties of the left parietal tool use region. Neuroimage.

[CR133] Pereira-Pedro AS, Bruner E, Gunz P, Neubauer S (2020). A morphometric comparison of the parietal lobe in modern humans and Neanderthals. J Hum Evol.

[CR134] Piercy M, Hécaen H, de Ajuriaguerra J (1960). Constructional apraxia associated with unilateral cerebral lesions—left and right sided cases compared. Brain.

[CR135] Possin KL, Laluz VR, Alcantar OZ (2011). Distinct neuroanatomical substrates and cognitive mechanisms of figure copy performance in Alzheimer’s disease and behavioral variant frontotemporal dementia. Neuropsychologia.

[CR136] Potì P (2005). Chimpanzees’ constructional praxis (*Pan paniscus*, *P. troglodytes*). Primates.

[CR137] Potì P, Langer J (2001). Spontaneous spatial constructions by chimpanzees (*Pan troglodytes*, *Pan paniscus*). Dev Sci.

[CR138] Potì P, Hayashi M, Matsuzawa T (2009). Spatial construction skills of chimpanzees (*Pan troglodytes* ) and young human children (*Homo sapiens sapiens*). Dev Sci.

[CR139] Povinelli D (2000). Folk Physics for Apes: The Chimpanzee’s theory of how the world works.

[CR140] Povinelli DJ, Reaux JE, Frey SH (2010). Chimpanzees’ context-dependent tool use provides evidence for separable representations of hand and tool even during active use within peripersonal space. Neuropsychologia.

[CR141] Quallo MM, Price CJ, Ueno K (2009). Gray and white matter changes associated with tool-use learning in macaque monkeys. Proc Natl Acad Sci.

[CR142] Quallo MM, Kraskov A, Lemon RN (2012). The activity of primary motor cortex corticospinal neurons during tool use by macaque monkeys. J Neurosci.

[CR143] Raimo S, Santangelo G, Trojano L (2021). The neural bases of drawing. A meta-analysis and a systematic literature review of neurofunctional studies in healthy individuals. Neuropsychol Rev.

[CR144] Ramayya AG, Glasser MF, Rilling JK (2010). A DTI investigation of neural substrates supporting tool use. Cereb Cortex.

[CR145] Ribas GC, Yasuda A, Ribas EC (2006). Surgical anatomy of microneurosurgical sulcal key points. Operat Neurosurg.

[CR146] Richtsmeier JT, Flaherty K (2013). Hand in glove: brain and skull in development and dysmorphogenesis. Acta Neuropathol.

[CR147] Russell C, Deidda C, Malhotra P (2010). A deficit of spatial remapping in constructional apraxia after right-hemisphere stroke. Brain.

[CR148] Shea JJ (2017). Occasional, obligatory, and habitual stone tool use in hominin evolution. Evol Anthropol.

[CR149] Silva-Gago M, Fedato A, Hodgson T (2021). Visual attention reveals affordances during Lower Palaeolithic stone tool exploration. Archaeol Anthropol Sci.

[CR150] Silva-Gago M, Ioannidou F, Fedato A (2022). Visual attention and cognitive archaeology: an eye-tracking study of palaeolithic stone tools. Perception.

[CR151] Stark A, Zohary E (2008). Parietal mapping of visuomotor transformations during human tool grasping. Cereb Cortex.

[CR152] Stiles J, Stern C (2001). Developmental change in spatial cognitive processing: complexity effects and block construction performance in preschool children. J Cogn Dev.

[CR153] Stiles-Davis J (1988). Developmental change in young children’s spatial grouping activity. Dev Psychol.

[CR154] Stiles-Davis J, Sugarman S, Nass R (1985). The development of spatial and class relations in four young children with right-cerebral-hemisphere damage: evidence for an early spatial constructive deficit. Brain Cogn.

[CR155] Stout D, Chaminade T (2007). The evolutionary neuroscience of tool making. Neuropsychologia.

[CR156] Stout D, Chaminade T (2012). Stone tools, language and the brain in human evolution. Philos Trans R Soc B.

[CR157] Stout D, Hecht E, Bruner E (2015). Neuroarchaeology. Human paleoneurology.

[CR158] Stout D, Hecht EE (2017). Evolutionary neuroscience of cumulative culture. Proc Natl Acad Sci USA.

[CR159] Stout D, Hecht E, Khreisheh N (2015). Cognitive demands of lower paleolithic toolmaking. PLoS ONE.

[CR160] Strauss H (1924). Über konstruktive Apraxie. pp. 65–84. Eur Neurol.

[CR161] Striem-Amit E, Vannuscorps G, Caramazza A (2017). Sensorimotor-independent development of hands and tools selectivity in the visual cortex. Proc Natl Acad Sci USA.

[CR162] Styrkowiec PP, Nowik AM, Króliczak G (2019). The neural underpinnings of haptically guided functional grasping of tools: an fMRI study. Neuroimage.

[CR163] Tan A, Tan SH, Vyas D (2015). There is more than one way to crack an oyster: identifying variation in Burmese long-tailed macaque (Macaca fascicularis aurea) stone-tool use. PLoS ONE.

[CR164] Umiltà MA, Escola L, Intskirveli I (2008). When pliers become fingers in the monkey motor system. Proc Natl Acad Sci.

[CR165] Valyear KF, Gallivan JP, McLean DA, Culham JC (2012). fMRI repetition suppression for familiar but not arbitrary actions with tools. J Neurosci.

[CR166] Van Lawick-Goodall J (1968). The Behaviour of Free-living Chimpanzees in the Gombe Stream Reserve. Animal Behav Monogr.

[CR167] Vereeken P (1961). Spatial development: constructive praxia from birth to the age of seven.

[CR168] Visalberghi E, Limongelli L (1994). Lack of comprehension of cause effect relations in tool-using capuchin monkeys (*Cebus apella*). J Comp Psychol.

[CR169] Visalberghi E, Tomasello M (1998). Primate causal understanding in the physical and psychological domains. Behav Proc.

[CR170] Visalberghi E, Trinca L (1989). Tool use in capuchin monkeys: distinguishing between performing and understanding. Primates.

[CR171] von Kleist K (1934). Gehirnpathologie. Brain.

[CR172] Weidenreich F (1941). The brain and its role in the phylogenetic transformation of the human skull. Trans Am Philos Soc.

[CR173] Wild HM, Heckemann RA, Studholme C, Hammers A (2017). Gyri of the human parietal lobe: volumes, spatial extents, automatic labelling, and probabilistic atlases. PLoS ONE.

[CR174] Wilson SAK (1908). A contribution to the study of apraxia with a review of the literature. Brain.

[CR175] Wynn T, Coolidge F (2003). The role of working memory in the evolution of managed foraging. Before Farming.

[CR176] Wynn T, Coolidge FL (2016). Archeological insights into hominin cognitive evolution. Evol Anthropol.

[CR177] Yoo K, Sohn WS, Jeong Y (2013). Tool-use practice induces changes in intrinsic functional connectivity of parietal areas. Front Hum Neurosci.

[CR178] Zilles K, Palomero-Gallagher N (2001). Cyto-, myelo-, and receptor architectonics of the human parietal cortex. Neuroimage.

[CR179] Zlatkina V, Petrides M (2014). Morphological patterns of the intraparietal sulcus and the anterior intermediate parietal sulcus of Jensen in the human brain. Proc R Soc B.

